# SnTox3 Acts in Effector Triggered Susceptibility to Induce Disease on Wheat Carrying the *Snn3* Gene

**DOI:** 10.1371/journal.ppat.1000581

**Published:** 2009-09-18

**Authors:** Zhaohui Liu, Justin D. Faris, Richard P. Oliver, Kar-Chun Tan, Peter S. Solomon, Megan C. McDonald, Bruce A. McDonald, Alberto Nunez, Shunwen Lu, Jack B. Rasmussen, Timothy L. Friesen

**Affiliations:** 1 Department of Plant Pathology, North Dakota State University, Fargo, North Dakota, United States of America; 2 USDA-ARS, Cereal Crops Research Unit, Red River Valley Agricultural Research Center, Fargo, North Dakota, United States of America; 3 Australian Centre for Necrotrophic Fungal Pathogens, Western Australian State Agricultural Biotechnology Centre, Division of Health Science, Murdoch University, Perth, Western Australia, Australia; 4 Plant Cell Biology, School of Biology, The Australian National University, Canberra Australian Capital Territory, Australia; 5 Plant Pathology Group, Institute of Integrative Biology, Swiss Federal Institute of Technology (ETH), Zurich, Switzerland; 6 USDA-ARS, Eastern Regional Research Center, Wyndmoor, Pennsylvania, United States of America; University of Melbourne, Australia

## Abstract

The necrotrophic fungus *Stagonospora nodorum* produces multiple proteinaceous host-selective toxins (HSTs) which act in effector triggered susceptibility. Here, we report the molecular cloning and functional characterization of the SnTox3-encoding gene, designated *SnTox3*, as well as the initial characterization of the SnTox3 protein. *SnTox3* is a 693 bp intron-free gene with little obvious homology to other known genes. The predicted immature SnTox3 protein is 25.8 kDa in size. A 20 amino acid signal sequence as well as a possible pro sequence are predicted. Six cysteine residues are predicted to form disulfide bonds and are shown to be important for SnTox3 activity. Using heterologous expression in *Pichia pastoris* and transformation into an avirulent *S. nodorum* isolate, we show that *SnTox3* encodes the SnTox3 protein and that *SnTox3* interacts with the wheat susceptibility gene *Snn3*. In addition, the avirulent *S. nodorum* isolate transformed with *SnTox3* was virulent on host lines expressing the *Snn3* gene. *SnTox3*-disrupted mutants were deficient in the production of SnTox3 and avirulent on the *Snn3* differential wheat line BG220. An analysis of genetic diversity revealed that *SnTox3* is present in 60.1% of a worldwide collection of 923 isolates and occurs as eleven nucleotide haplotypes resulting in four amino acid haplotypes. The cloning of *SnTox3* provides a fundamental tool for the investigation of the *S. nodorum*–wheat interaction, as well as vital information for the general characterization of necrotroph–plant interactions.

## Author Summary

The necrotrophic fungus *Stagonospora nodorum* produces multiple toxins that are effective in causing disease on wheat. Here, we report the characterization of the SnTox3-producing gene, designated *SnTox3*, as well as the initial characterization of the SnTox3 protein. In order to verify the action of this toxin, we expressed *SnTox3* in yeast to show that *SnTox3* encodes the SnTox3 protein which interacts directly or indirectly with the product of the corresponding wheat susceptibility gene *Snn3*. Transformation of a non pathogenic *S. nodorum* isolate with *SnTox3* indicated that expression of the SnTox3 gene is sufficient to render an avirulent isolate virulent in the presence of *Snn3*. *SnTox3* disruption mutants are deficient in the production of SnTox3 and consequently are avirulent on the *Snn3* differential wheat line BG220. *SnTox3* is present in approximately 60% of a worldwide collection of 923 isolates. The cloning of *SnTox3* provides a critical tool for the investigation of the *S. nodorum*–wheat interaction, but also significantly adds to a necrotrophic effector system that is an exciting contrast to the biotrophic effector models that have been intensively studied.

## Introduction

Diseases caused by necrotrophic pathogens are believed to be distinctly different from those caused by biotrophs where the pathogen requires a living host to grow and sporulate. The interaction between biotrophic pathogens and their hosts is controlled via the interaction of pathogen effector molecules which, if recognized by the corresponding resistance gene product, results in localized programmed cell death (PCD) and activation of plant defense which leads to a resistant or incompatible interaction. This interaction has been referred to as effector triggered immunity (ETI) [1]. In contrast, necrotrophic pathogens induce host cell death during infection. Host-selective toxins (HSTs) are molecules produced by some necrotrophic fungi that induce a necrotic reaction and promote a susceptible/compatible interaction in the host [2]. Most HSTs are small secondary metabolites and their production is under the control of complex genetic and enzymatic pathways [3]. But several proteinaceous HSTs have been identified more recently [4]–[8].

*Stagonospora nodorum*, the causal agent of Stagonospora nodorum blotch (SNB), is a necrotrophic fungus that causes major yield losses worldwide by infecting the leaves and glumes of wheat [9]–[11]. SNB resistance is quantitatively inherited with little obvious evidence of an isolate-specific relationship between the pathogen and the host [12]. Early research on SNB indicated that resistance was mostly controlled by multiple genes located on chromosomes throughout the genome, with each showing minor association with disease [13],[14].

We recently identified four proteinaceous HSTs from *S. nodorum* and showed they play an important role in disease development by interacting with corresponding host sensitivity/susceptibility gene products [14]. One of these HSTs, ToxA, is a small proteinaceous HST produced by approximately 40% of *S. nodorum* isolates worldwide and is present in multiple forms in *S. nodorum* [15]. ToxA interacts with the product of the wheat gene *Tsn1* [16] and fungal strains carrying *ToxA* are significantly more virulent on wheat lines carrying *Tsn1*. *ToxA* is also expressed by the tan spot pathogen *Pyrenophora tritici-repentis* [4] and induces necrosis in a process which involves uptake into the host cytoplasm, translocation to the chloroplast and disruption of photosynthesis [17].

SnTox1 and SnTox2 also appear to be small proteins, though their encoding genes have not yet been cloned [18],[19]. Preparations of SnTox1 and SnTox2 induce necrosis on wheat lines carrying the toxin sensitivity genes *Snn1* and *Snn2*, and fungal isolates producing these toxins are more virulent on wheat lines carrying *Snn1* and *Snn2*, respectively. The presence of these toxins also varies between isolates and the strain Sn79-1087 appears to produce no toxins at all and is avirulent on all tested wheat lines [15].

We previously characterized the reaction of a necrosis-inducing activity that defines a fourth host locus called *Snn3*. Culture filtrate fractions of *S. nodorum* strain Sn1501 were used to define a QTL on chromosome 5BS in a cross between wheat lines BR34 and Grandin. SnTox3 is proteinaceous and was reported to be 10–30 kDa in size [20]. QTL analysis showed that the SnTox3-*Snn3* interaction contributed significantly to disease development [20].

Here we report the molecular cloning of the SnTox3-encoding gene *SnTox3*, and show that SnTox3 plays an important role in disease by interacting directly or indirectly with the product of the wheat sensitivity gene *Snn3*. The cloning of *SnTox3* provides a critical tool for the molecular and biochemical characterization of the host-pathogen interaction in the *S. nodorum*-wheat pathosystem.

## Results

### Identification of the SnTox3-encoding gene

**Figure 1:**
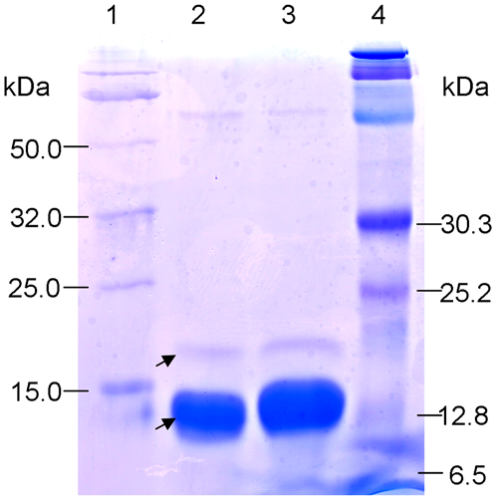
SDS-PAGE image of a size exclusion chromatography fraction containing SnTox3 activity. Lane 1. Genescript Smart His-tagged protein standard; Lane 2 and 3, duplicate protein samples of the size exclusion chromatography fraction 17-12 (Figure S1B); Lane 4. Bio-Rad pre-stained protein standard. The molecular weight (kDa) of each protein is labeled and the arrows indicate the two protein bands that were excised from the gel. The larger band is estimated at 18 kDa and contains SnTox3.

Progeny line BG220 from the BR34 x Grandin recombinant inbred population was previously identified as a SnTox3 differential line [20] and was used in identifying and characterizing this toxin. Ion exchange and size exclusion chromatography was used for the initial purification of BG220-reactive material as previously described [20]. A large peak and three smaller peaks were observed in size exclusion chromatography (Figure S1); the large peak was associated with SnTox3 activity and was analyzed by SDS-PAGE (Figure 1). The protein gel was visualized as containing a light band and a dark band within the size range of 6.5 to 32.0 kDa. The light band was estimated at 18.0 kDa and the darker band was estimated at 13.0 kDa. Mass spectra obtained from the two bands was used to search the *S. nodorum* protein database [11] leading to the identification of two predicted proteins, SNOG_08981 and SNOG_16063, corresponding to the light and dark protein bands in the gel, respectively.

**Figure 2:**
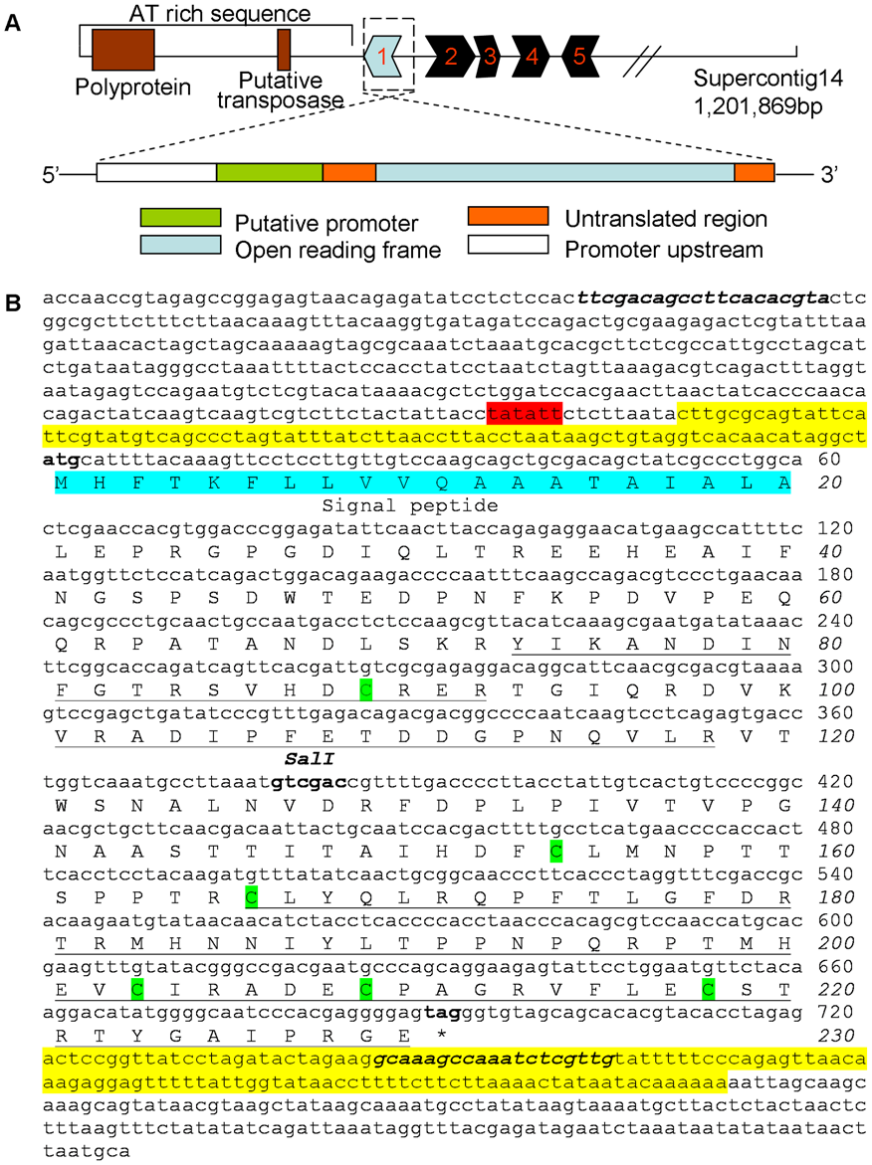
Genomic location, gene structure and nucleotide and amino acid sequence of *SNOG_08981*. A. The genomic location and gene structure of *SNOG_08981*. Top: Supercontig14 was schematically drawn to show one end containing *SNOG_08981* (Gene 1 - light blue-color filled arrow) as well as the four other SNOG genes (2, 3, 4 and 5, black color filled arrows) present in the flanking region. A large amount of AT rich sequence closely flanking *SNOG_08981* was labeled that contains a polyprotein gene and a putative transposase gene. Below: A close up of a 1.6 kb genomic region (dash boxed) indicates a single exon, the 5′- and 3′- UTR, and the putative promoter region. B. Nucleotide sequence of a 1.6 kb genomic region of *SNOG_08981* (*SnTox3*) and the deduced amino acid sequence of the SNOG_08981 protein (SnTox3). The underlined peptide sequence was detected in mass spectrometry. The amino acids highlighted in blue indicate the predicted signal peptide. The six cysteine residues highlighted in green are predicted to form 3 disulfide bonds. The bold DNA sequence indicates the start codon (ATG), stop codon (TAG) and the *Sal*I restriction site. DNA sequence highlighted in yellow is UTR and the red highlighted region is a putative TATA box. Bold italicized sequence indicates the primer sites used to amplify the genomic region for transformation.

PCR amplification revealed that *SNOG_16063* was present in all strains tested whereas *SNOG_08981* was absent in the avirulent isolate Sn79-1087 (Figure S2). Since Sn79-1087 did not produce BG220-reactive material, this indicated that *SNOG_08981* could correspond to the SnTox3 encoding gene. Further analysis of the MALDI-TOF/TOF MS and MS/MS spectra of the trypsinized peptides identified a total of 44% of the predicted immature SNOG_08981 protein sequence (see Figure S3 for mascot search results of SNOG_08981). This included the majority of the C-terminal region but did not include the first 20 amino acids predicted to be the signal sequence nor did it account for amino acids 21–72 of the N-terminus (Figure 2).

### Genomic context of *SNOG_08981*

The predicted *SNOG_08981* gene was located on the end of supercontig14 [11] which is ∼1.2 Mb in length and is currently estimated to harbor 455 genes (Figure 2A, [11], Hane and Oliver, unpublished). Between *SNOG_08981* and the end of the contig is a ∼10 kb region of AT-rich sequence. No additional genes are predicted in this AT-rich region except a polyprotein and a putative transposase (Figure 2A). Some of the AT-rich sequence is annotated as part of the Elsa family of degraded retrotransposons [11]. Of the four genes upstream of *SNOG_08981*, two of them are functionally conserved, and the other two are hypothetical genes based on the genome sequence annotation [11].

### *SNOG_08981* contains no introns and has no identified homologs in other fungi

The full-length cDNA of the *SNOG_08981* gene was identified by reverse-transcription and rapid amplification of cDNA ends (RACE). The gene comprises an 80 bp 5′ UTR, a 146 bp 3′ UTR and a single exon of 693-nucleotides encoding a protein with 230 amino acids (Figure 2A and B). A putative TATA box was located 95 bp upstream of the start codon whereas no obvious CAAT box was identified (Figure 2B).

The *SNOG_8981* cDNA sequence identified was different from that found in the genome database (http://www.broad.mit.edu/annotation/genome/stagonospora_nodorum). The genome sequence archives at this region were retrieved to perform re-assembly and the difference was shown to be due to an error in the sequence assembly. The reassembled sequence fully matched our cDNA sequence. The corrected *SNOG_08981* sequence was submitted to NCBI as Genbank accession number FJ823644.

Using the *SNOG_08981* ORF sequence in BLASTN or BLASTX searches of the NCBI nr database, no significant similarity to sequences from other organisms was detected. The *S. nodorum* hypothetical protein, SNOG_10812, was the only hit in a BLASTX search with a score at 67.0 bits and an E-value at 1e-09. Other hits were obtained but were identified at E-values greater than 0.1.

### Southern analysis of *SNOG_08981* in *S. nodorum* strains

**Figure 3:**
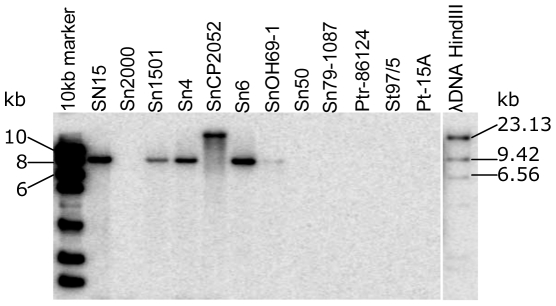
Southern analysis of *SNOG_08981* in *S. nodorum* and related fungi. Southern hybridization was performed using a probe from the full length *SNOG_08981* ORF as well as λDNA. Size ladders include Hyperladder I and λDNA *Hin*dIII fragments. The isolate designations starting with Sn or SN are *S. nodorum* isolates, and Ptr86-124, St975, and Pt-15A, are isolates of *Pyrenophora tritici-repentis*, *Mycosphaerella graminicola* and *P. teres*, respectively.

Using the full-length gene as a probe, Southern analysis of genomic DNA from a small sub-set of *S. nodorum* isolates indicated that the *SNOG_08981* gene was present as a single copy gene in six of the eight *S. nodorum* virulent isolates, but absent in the other two virulent isolates, as well as the avirulent isolate (Figure 3). All *SNOG_08981*-containing isolates showed the same size hybridizing band as SN15 except for the Danish isolate SnCP2052 where a larger band, suggests there is sequence variation flanking *SNOG_08981* in this isolate (Figure 3).

The detectable toxin activity from different *S. nodorum* isolates was summarized in Table 1. The six *S. nodorum* isolates found containing *SNOG_08981* were also known to produce SnTox3 in culture (Table 1). The *SNOG_08981* probe did not hybridize to the virulent isolates Sn2000 [18],[21], or Sn50; accordingly, culture filtrates from these strains were also unable to induce necrosis on BG220 indicating Sn2000 and Sn50 do not produce SnTox3 (Table 1). Sn2000 has been used in QTL analysis in the BG population and no QTL associated with disease susceptibility was identified at the *Snn3* locus [21],[22]. However, these two isolates produce SnToxA, SnTox1 and/or other toxins, which make them virulent on wheat lines carrying either *Tsn1* or *Snn1* or both [15],[18],[21] (unpublished data).

**Table 1:** Fungal isolates or strains used in the yeast expression, *SnTox3* transformation, *SnTox3* disruption, protein haplotypes analysis, and Southern hybridization.

Isolate or strain¶	Origin/Resource	Toxin detectable in the culture†				*SnTox3* ‡	References
		SnToxA	SnTox1	SnTox2	SnTox3		
Sn4	ND, USA	+	+	+	+	+	Friesen, et al. unpublished
Sn15	Australia	+	?	+	+	+	[15]
Sn1501	OH, USA	−	?	+	+	+	[20]
Sn50	ND, USA	+	?	+	−	−	Friesen, et al. unpublished
Sn2000	ND, USA	+	+	−	−	−	[18],[21]
SnCP2052	Denmark	+	?	+	+	+	Friesen, et al. unpublished
Sn6	ND, USA	+	?	+	+	+	[19]
SnOH69-1	OH, USA	−	?	+	+	+	Friesen, et al. unpublished
ARKW40	AK,USA	?	?	?	?	+	[39]
KZ3.4.10	Kazakhstan	?	?	?	?	+	[39]
Sn79-1087	ND,USA	−	−	−	−	−	[15]
Sn79+SnTox3A	This study	−	−	−	+	+	This study
Sn79+SnTox3B	This study	−	−	−	+	+	This study
Sn1501ΔSnTox3A	This study	−	?	+	−	−	This study
Sn1501ΔSnTox3B	This study	−	?	+	−	−	This study
Sn1501Ect	This study	−	?	+	+	+	This study

**¶:** Sn1501 and SnOH69-1 were provided by Dr. Pat Lipps, Ohio State University; the avirulent isolate Sn79-1087 was provided by Dr. Joseph Krupinsky, retired, Northern Great Plains Research Laboratory USDA-ARS, Mandan, ND, USA.

**†:** Toxin activity is detectable (+), not detectable (−) or not tested (?).

**‡:** Identifies the presence (+) or absence (−) of the *SnTox3* (*SNOG_08981*) gene in isolates or strains.

### Gene expression of *SNOG_08981* is maximized at the early stages of infection

**Figure 4:**
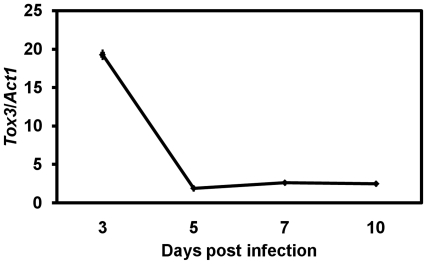
Expression pattern of *SnTox3 in planta*. The expression level of *SnTox3* was examined and compared to that of the *Act1* gene at 3, 5, 7 and 10 days after inoculation. The x axis shows the number of days post-infection. The y axis represents relative gene expression levels normalized to *Act1*. Standard error bars are shown.

*SNOG_08981* expression was examined at three, five, seven, and ten days post-infection on the SnTox3 sensitive wheat c.v. ‘Amery’ using quantitative real time RT-PCR. The transcription levels of *SNOG_08981* were maximal at three days post infection coinciding with the hyphal proliferation and the onset of lesion development on the leaf (Figure 4). Transcription levels were significantly reduced from five days post-infection which coincided with a transition from hyphal proliferation to asexual sporulation. This observation was essentially confirmed with microarray analysis of the *S. nodorum* transcriptome at the same post-infection time points (data not shown).

### Heterologous expression of *SNOG_08981* in *Pichia pastoris*

**Figure 5:**
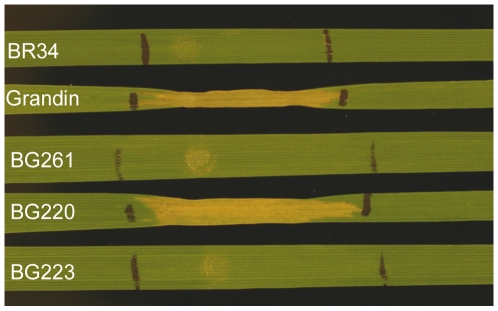
Toxin bioassay of the *Pichia pastoris* X33 strain transformed with *SNOG_08981*. Leaves of toxin differential and parental lines, including BR34, Grandin, BG261, BG220 and BG223 were infiltrated with culture filtrate from *P. pastoris* X33 transformed with *SNOG_08981*. Wheat lines containing *Snn3* (Grandin and BG220) were sensitive whereas lines not containing *Snn3* (e.g. containing *snn3*) (BR34, BG261, and BG223) were insensitive.

A yeast expression construct containing the full-length cDNA of *SNOG_08981* was transformed into *P. pastoris*. The culture filtrate from positive yeast clones were harvested and used to infiltrate the toxin differential lines BG261 (SnToxA-sensitive; *Tsn1*), BG223 (SnTox2-sensitive; *Snn2*), and BG220 (SnTox3-sensitive; *Snn3*) as well as parental lines BR34 and Grandin [15],[19],[20]. The culture filtrates produced a necrotic reaction on Grandin (*Snn3*) and BG220 (*Snn3*), but not on BR34 (*snn3*), BG261 (*snn3*) and BG223 (*snn3*) (Figure 5). The culture filtrate from the yeast transformed with an empty vector (negative control) did not induce necrosis on any of the differential lines (Figure S4). All BG lines sensitive to partially purified SnTox3 [20] were also sensitive to *SNOG_08981* transformed yeast culture filtrates, and thereby the sensitivity was mapped to the *Snn3* locus on wheat chromosome arm 5BS in the BG population as previously described [20]. This strongly indicates that *SNOG_08981* is the SnTox3-encoding gene and therefore we designated it as *SnTox3*.

### *SnTox3* renders an avirulent *S. nodorum* strain virulent in host lines containing *Snn3*

**Figure 6:**
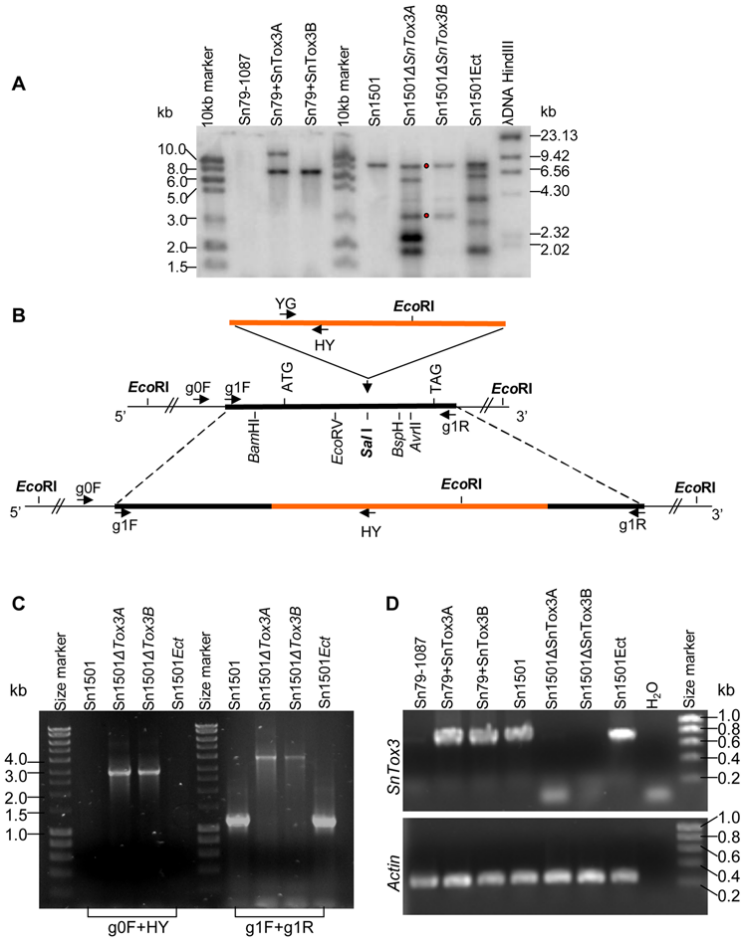
Molecular characterization of *SnTox3* transformation in Sn79-1087 and disruption in Sn1501. A. Verification of the *SnTox3* transformation into Sn79-1087 and disruption in Sn1501 using Southern analysis. Genomic DNA from the avirulent isolate Sn79-1087 and its two *SnTox3* transformed strains Sn79+SnTox3A and Sn79+SnTox3B were digested with *Xho*I. The virulent *SnTox3* containing isolate Sn1501 and its two *SnTox3*-disrupted strains Sn1501ΔSnTox3A and Sn1501ΔSnTox3B as well as a strain with an ectopic integration, Sn1501Ect, were digested with *Eco*RI. The blot was probed with a full length *SnTox3* cDNA. The two red dots indicate the bands of 7.5 kb and 3.0 kb specifically present in the *SnTox3*-disrupted strains (see Figure 7B). A 7.9 kb band is present in the wild type and ectopic strain. More ectopic insertions were detected by southern in Sn1501ΔSnTox3A and Sn1501Ect, compared to Sn1501ΔSnTox3B. B. *SnTox3* disruption strategy in Sn1501. The *SnTox3* gene in Sn1501 was disrupted by insertion of the hygromycin B resistance gene (orange bar) into a *Sal*I restriction site located in the center of the *SnTox3* gene. A ∼1.2 kb genomic region of *SnTox3* (black bar) amplified with primers 8981g1F_XbaI and 8981g1R_XbaI was cloned and then linearized with *Sal*I. The linearized vector was re-ligated with a *cpc-1*:*HYG* :*tryptophan c* cassette that was released from the pLP605KO vector [51] using the restriction enzyme *Xho*I. The resulting vector was linearized via *Xba*I before transformation into Sn1501 protoplasts. The disruption of *SnTox3* can be identified using primers 8981g0F with HY and verified by primers 8981g1F and 8981g1R. C. PCR screening and verification of *SnTox3*-disrupted mutants in Sn1501. The primer 8981g0F (g0F) located outside of the ∼1.2 kb region along with primer HY amplify a ∼2.9 kb fragment in strains which have *SnTox3* disrupted by the insertion. No amplification is observed in wild type and ectopics. The primers 8981g1F1 (g1F) and 8981g1R (g1R) amplify a ∼1.2 kb fragment in the wild type and ectopic strains indicating the *SnTox3* gene remains intact, while they amplify a ∼3.8 kb fragment (∼1.2 kb of *SnTox3* region plus ∼2.6 kb of *cpc-1*:*HYG* :*tryptophan c* cassette) in the *SnTox3*-disrupted strains. D. RT-PCR verification of different mutated strains. The primers 8981cF and 8981cR which amplify the *SnTox3* ORF region were used to test the presence of transcripts of *SnTox3* in different isolates and genetically modified strains including Sn79-1087 and its two transformed *SnTox3+* strains (Sn79+SnTox3A and Sn79+SnTox3B) and Sn1501 and its two *SnTox3*-disrupted strains Sn1501ΔSnTox3A and Sn1501ΔSnTox3B along with the ectopic transformant Sn1501Ect. The *S. nodorum* actin gene [49] was used as an internal control and water was used as a PCR negative control.

Culture filtrate of *S. nodorum* isolate Sn79-1087, which was isolated from a wild grass, produces no necrosis on any wheat line we have studied. Furthermore it is completely avirulent to all wheat lines that have been tested. Expression of *ToxA* in Sn79-1087 rendered it pathogenic on BG261 and made it capable of producing active ToxA [15]. A fungal transformation construct containing the genomic region of the *SnTox3* gene and the hygromycin B resistance gene was transformed into Sn79-1087. Two putative transformants, Sn79+SnTox3A and Sn79+SnTox3B were selected and analyzed for *SnTox3* gene integration, toxin production, and virulence change compared to the wild type. Southern analysis using the *SnTox3* full-length cDNA as a probe showed that the Sn79+SnTox3A and Sn79+SnTox3B transformants harbored at least two and one copies of *SnTox3*, respectively. Both transformants contained the same size restriction fragment (∼7.0 kb) suggesting that one of the *SnTox3* integrations was present in the same place in the Sn79-1087 genome (Figure 6A). RT-PCR also confirmed the transcription activity of *SnTox3* in the two transformants (Figure 6D).

**Figure 7:**
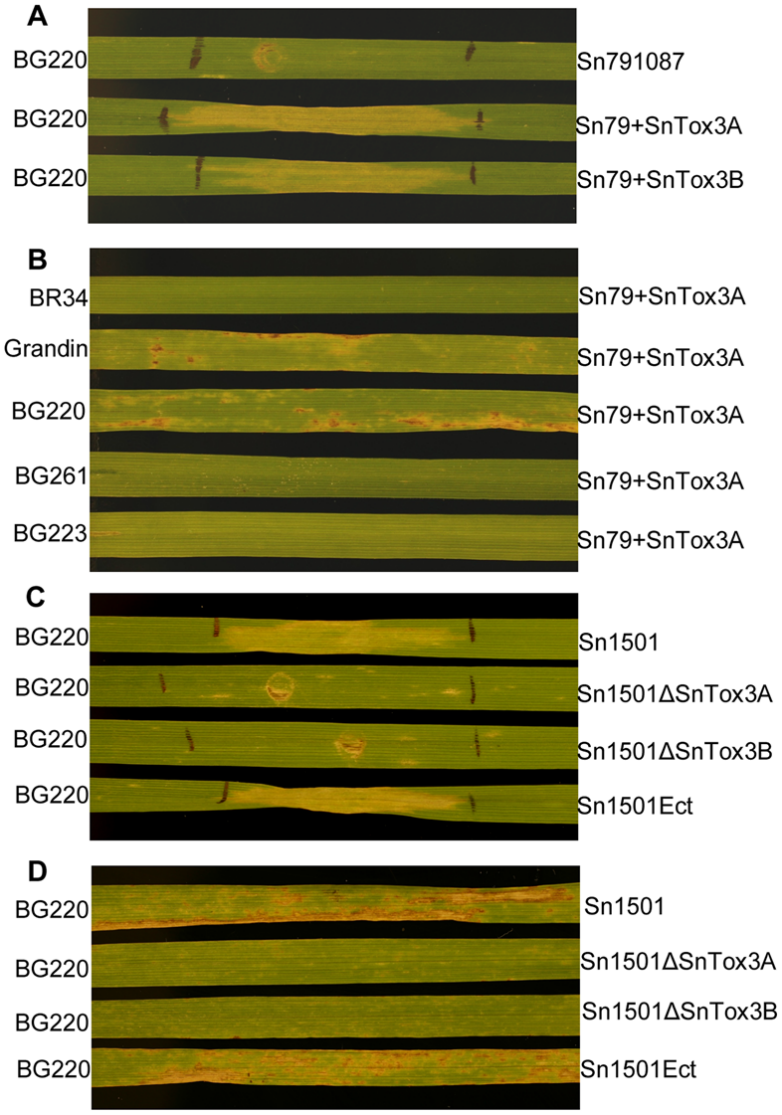
Toxin bioassay and virulence analysis of the *SnTox3* genetically modified strains. A. Toxin bioassay of Sn79-1087 with its two transformed *SnTox3*+ strains Sn79+SnTox3A and Sn79+SnTox3B. Leaves of BG220 (SnTox3 differential line) were infiltrated with culture filtrates of Sn79-1087 wild type, Sn79+SnTox3A and Sn79+SnTox3B and photographed 3 days after infiltration showing culture filtrates from the wild type isolate did not induce necrosis while the strains transformed with *SnTox3* (Sn79+SnTox3A and Sn79+SnTox3B) induced necrosis. B. Virulence of the *SnTox3* transformed strain Sn79+SnTox3A. Conidia from Sn79+SnTox3A were inoculated onto BR34, Grandin, BG220, BG261 and BG223 showing this strain was virulent on Grandin and BG220 which harbors *Snn3*, while it remained avirulent on BR34, BG261, and BG223 which harbors *snn3*. C. Toxin bioassay of *SnTox3* disrupted strains. Leaves of BG220 (*Snn3*) were infiltrated with culture filtrate of Sn1501 wild type, Sn1501ΔSnTox3A, Sn1501ΔSnTox3B, and the ectopic strain Sn1501Ect and photographed 3 days after infiltration. Culture filtrates from the wild type and ectopic strains were able to induce necrosis while those from the two *SnTox3*-disrupted strains were unable to induce necrosis. D. Virulence comparison of Sn1501 wild type and the *SnTox3* disrupted strains on BG220 (*Snn3*). Inoculation of conidia from Sn1501 wild type, Sn1501ΔSnTox3A, Sn1501ΔSnTox3B and Sn1501Ect were inoculated onto BG220 (*Snn3*) showing the loss of virulence of *SnTox3* disrupted strains on the SnTox3 differential line BG220.

Culture filtrates of Sn79-1087, Sn79+SnTox3A and Sn79+SnTox3B were infiltrated into BG220. Necrosis was not induced using the wild type Sn79-1087 strain, whereas the two transformants caused a strong necrotic reaction (Figure 7A). Culture filtrates from the two transformants were also infiltrated into all 118 RI lines in the BG mapping population to verify that sensitivity mapped to the *Snn3* locus. As expected, sensitivity to culture filtrates of both transformants in the BG population was conferred by a single gene and mapped to chromosome arm 5BS at the *Snn3* locus [20]. This provides additional evidence that *SNOG_08981* is responsible for the production of SnTox3.

**Figure 8:**
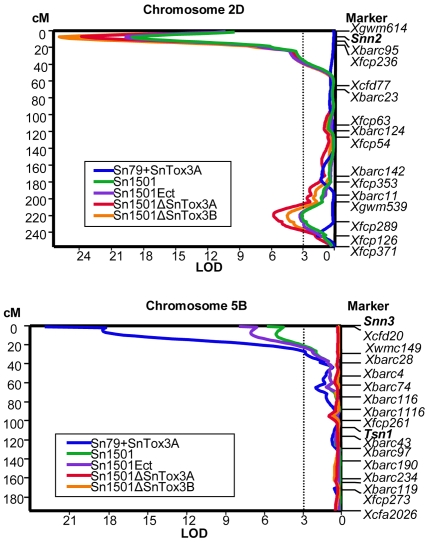
Interval map of chromosome 2D (Top) and 5B (bottom). The map shows QTL of susceptibility associated with *Snn2* and *Snn3* loci in the BG population after being inoculated with genetically modified fungal strains. Strains are depicted by different colors as indicated. A centiMorgan scale is on the left of the map and markers are shown in their relative position along the right. An LOD scale is shown along the x axis, and the critical LOD threshold of 3.0 is indicated by the dotted line.

To test if the addition of *SnTox3* changes the specificity of Sn79-1087, the conidia from strain Sn79+Tox3A were harvested and used to inoculate the BG differential lines as well as BR34 and Grandin. This isolate caused typical *S. nodorum* lesions on Grandin and BG220 which contain *Snn3* (Figure 7B). No visible lesions were observed on the *snn3* lines BR34, BG261 and BG223. This isolate was further inoculated onto the entire BG population and QTL analysis showed that the *Snn3* locus explained the majority of the variation in susceptibility (59%) to the fungus and no other QTL was identified (Figure 8). From these data, we can conclude that the addition of the *SnTox3* gene is sufficient to change an avirulent *S. nodorum* strain into a virulent strain by inducing effector triggered susceptibility on host lines containing *Snn3*.

### *SnTox3*-disrupted virulent isolates are reduced in virulence specific to *Snn3* wheat lines

*SnTox3* was disrupted in Sn1501, a strain which is deficient in the *SnToxA* gene but contains *SnTox3* (Figure 3) and produces SnTox3 (Table 1). As *SnTox3* is closely linked to a large region of retrotransposon-related elements (Figure 2A), a gene replacement knockout strategy was unavailable. Instead we inserted the hygromycin B resistance gene (*HYG*) cassette into the *SnTox3* coding region to construct a gene disruption vector (see Material and Methods for vector construction). In this vector, the HYG cassette was flanked by ∼700 bp of *SnTox3* 5′ region at one end and ∼500 bp of *SnTox3* 3′ region at the other (Figure 6B). Two sets of primers: 8981g0F and HY, 8981g1F and 8981g1R were used to screen and verify transformants (see Figure 6B for primer locations and Table S1 for sequence). Three out of 68 resulting strains contained the disrupted *SnTox3* cassette, whereas the remaining 65 transformants harbored ectopic integrations. Two of the *SnTox3*-disrupted strains, designated Sn1501ΔTox3A and Sn1501ΔTox3B, and one ectopic strain, designated Sn1501Ect, were selected for further analysis. PCR, Southern blotting and RT-PCR confirmed the gene-disruption (Figure 6A, 6C and 6D).

The culture filtrates of four strains were tested on the SnTox3 differential line BG220 and the results showed that the wild type (Sn1501) and ectopic transformants (Sn1501Ect) were able to produce SnTox3. The two strains harboring the mutated *SnTox3* did not produce active SnTox3 *in vitro*, suggesting that SnTox3 was non-functional in these two mutants (Figure 7C). Sn1501 wild type, Sn1501ΔSnTox3A, Sn1501ΔSnTox3B, and Sn1501Ect were inoculated onto the SnTox3 differential line BG220. Sn1501 and Sn1501Ect induced typical lesions on BG220, whereas only small white flecks were present without any visible lesions on the leaves inoculated with Sn1501ΔSnTox3A and Sn1501ΔSnTox3B, indicating the mutants were avirulent on BG220 (Figure 7D). Compared to the wild type, the two *SnTox3*-disrupted strains also showed decreased virulence toward the parental line Grandin which also contains *Snn3*; however, *SnTox3* disruption did not change the reaction towards parental line BR34 or the SnToxA and SnTox2 differential lines BG261 and BG223 respectively, which do not harbor *Snn3* (Figure S5).

The four strains Sn1501, Sn1501ΔSnTox3A, Sn1501ΔSnTox3B, and Sn1501Ect were also inoculated onto the entire BG population in order to quantify the effect of the SnTox3-*Snn3* interaction using QTL analysis. For wild type Sn1501, as previously reported [20], significant QTLs were detected on the distal end of chromosome 2DS and 5BS which corresponded to *Snn2* (SnTox2 sensitivity) and *Snn3*, respectively. The *Snn2* and *Snn3* loci explained 46% and 10% of the variation in disease, respectively. The same two QTLs with similar effects (46% and 13%) were found to be associated with susceptibility to the Sn1501Ect strain. For the two *SnTox3*-disrupted strains Sn1501ΔSnTox3A and Sn1501ΔSnTox3B, QTL analysis showed that the *Snn2* locus explained 60 and 64% of the variation, respectively, and the effects of the *Snn3* locus were not significant (Figure 8) showing that SnTox3 specifically interacts with *Snn3* and the loss of SnTox3 does not negatively affect the SnTox2-*Snn2* interaction. Together, these results demonstrate that *SnTox3* codes for the SnTox3 protein which plays a significant role in disease on lines carrying the wheat HST-sensitivity gene *Snn3*.

### Preliminary characterization of the SnTox3 protein

The predicted SnTox3 pre-protein contains 230 amino acids with a calculated mass of 25.85 kDa. SnTox3 activity was detected in culture filtrates and both SignalP 3.0 (http://www.cbs.dtu.dk/services/SignalP/) and WolfPsort (http://wolfpsort.org/) signal peptide prediction software predicted that the first 20 amino acids are a signal peptide (Figure 2) necessary for the secretion of the protein.

**Figure 9:**
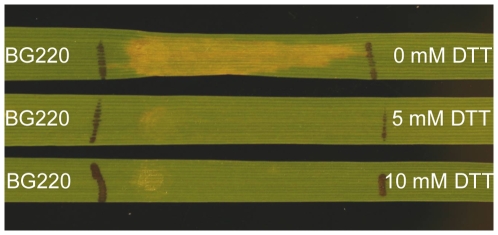
SnTox3 activity is sensitive to Dithiothreitol (DTT). Leaves of BG220 were infiltrated with culture filtrates of *P. pastoris* expressing SnTox3 that had been treated for 2 h with 0 mM, 5 mM and 10 mM DTT.

SnTox3 contains six cysteine residues. Using DISULFIND at the website of PredictProtein (http://www.predictprotein.org), six cysteine residues in the SnTox3 protein were predicted to form three disulfide bonds with a confidence level of 9 (0–9 scale) (Figure 2B). The best connectivity pattern based on the prediction is C89–C209, C154–C203, and C166–C218. These disulfide bridges may play an important role in the stability of the protein. Dithiothreitol (DTT) treatment of *P. pastoris* SnTox3 culture filtrates eliminated necrotic activity (Figure 9) suggesting the importance of at least one of the disulfide bonds in SnTox3 activity.

### Diversity and function of *SnTox3* in global *S. nodorum* populations

A total of 923 samples were collected from eight major geographical regions and were screened for presence/absence of *SnTox3* using PCR. The *SnTox3* deletion frequencies ranged from 3.51% in Australia to 61.7% in the Middle East, with a global deletion frequency of 39.9% (Table S2). Dot Blot Hybridization, with a subset of isolates, confirmed that isolates with no PCR amplicon did not contain a copy of *SnTox3*

**Figure 10:**
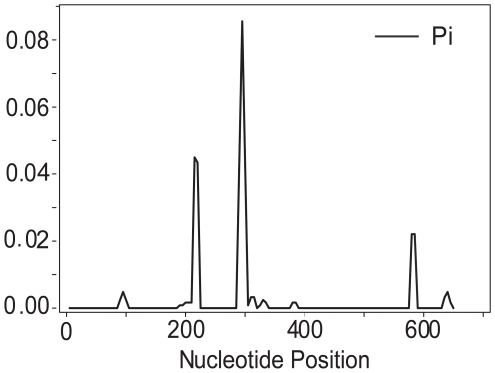
Calculation of nucleotide diversity (Pi) at the *SnTox3* coding region. The calculations were done using a sliding window of size 10 with a step size of 5. Pi values of 0 indicate conserved regions of the gene for all 245 sequenced isolates.

**Figure 11:**
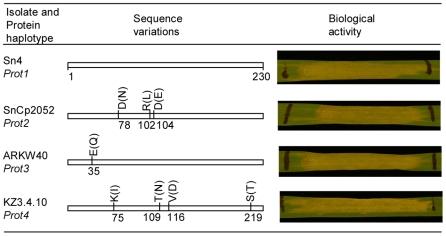
The four protein variants of SnTox3 and their biological activity. The different haplotypes of SnTox3 were amplified from the corresponding representative isolates and expressed in *P. pastoris*. The unfilled bars represent the complete SnTox3 protein and the number under the bar shows the position of each sequence variation indicated by pairs of amino acids (the first is the protein variant 1 and the substitution is in parenthesis). The biological activity of yeast clones expressing each protein haplotype is shown to the right.

Sequence diversity of *SnTox3* was assessed by sequencing the gene for 245 isolates. Eleven nucleotide haplotypes were identified which encoded four amino acid sequences (Table S2). Nucleotide diversity was estimated using the pair-wise difference measurement Pi [23] and shown in Figure 10. There were 9 synonymous and 8 non-synonymous substitutions across 654 nucleotide sites. Isolates Sn4, SnCP2052, ARKW40, and KZ3.4.10 were used to amplify and clone *SnTox3* alleles representing protein variants 1, 2, 3, and 4, respectively. The amino acid differences for the four protein variants are shown in Figure 11. Protein variant 1 composed 66% of the sequenced isolates followed by protein variant 2 which composed 31% of the sequences. Protein variant 1 was used in the *P. pastoris* expression experiments described earlier, but the other three protein variants were also heterologously expressed in *P. pastoris* and were all found to induce necrosis on the SnTox3 differential line BG220 (Figure 11, Table S2).

## Discussion

Until recently, the *S. nodorum*-wheat pathosystem was thought to be based on the interaction of a suite of non-specific toxins and cell wall degrading enzymes [24]. No specific variation in virulence on the host was recognized. Resistance was quantitative and defined as a plethora of mostly weak and environment-specific QTL [14]. As a result, breeding for disease resistance was based on simple phenotypic assessment; no molecular markers were in use.

The assembly of critical tools including functional genomics, host mapping populations, and the genome sequence has allowed the role of pathogen-produced effectors (HSTs) to be revealed. We previously characterized the SnToxA-*Tsn1* interaction in which some isolates produced different variants of ToxA that interact directly or indirectly with the product of the wheat gene *Tsn1* to produce a necrotic reaction that benefits the pathogen [15]. Three additional toxin/receptor interactions have been partially characterized [18]–[20]. Together, these findings have opened up a rational route to the genetic control of this disease [14],[25] where pathogen produced effectors (HSTs) interact with dominant host gene products. But in contrast to effector triggered immunity (ETI) found in many biotrophic systems [26], the end result in the *S. nodorum*-wheat interaction is effector triggered susceptibility (ETS).

Proteomic analysis of purified active fractions identified the candidate gene *SNOG_08981*. A range of tests presented here provided convincing and comprehensive evidence that the candidate gene *SNOG_08981* corresponds to *SnTox3* and that addition or ablation of SnTox3 changes the specificity of the corresponding fungal strains on the differential line harboring the host gene *Snn3*.

*SnTox3* is an intron-free 693 nucleotide gene encoding a predicted 230 amino acid immature protein. MS and MS/MS spectra of the digested protein identified 44% of the total sequence, including amino acids from position 73 to the C-terminus. The first 20 amino acids make up a predicted signal sequence that likely serves as a signal peptide for secretion. This is consistent with the detection of SnTox3 activity in the culture filtrate of fungal isolates that harbor *SnTox3*. As amino acids 21 through 72 are not accounted for by MS and also SnTox3 was observed as an ∼18 kDa protein on SDS-PAGE, it is likely that SnTox3 is a pre-pro protein similar to ToxA and that the mature protein requires the cleavage of an additional N-terminal region including part or all of amino acids 21 to 72. It is interesting to note that immediately before the first peptide identified by mass spectrometry, there are four residues (LSKR) similar to an LRKR sequence in the effector protein Six1 which is predicted as a Kex2-like protease recognition site [27]. The calculated molecular weight for the remainder of the protein is 17.88 kDa similar to what we observed for SnTox3 using SDS-PAGE. Additional work is ongoing to verify the N-terminal region of the mature protein.

SnTox3 is a unique protein and presently has no obvious homology to other proteins present in current protein databases other than *S. nodorum*. Many pathogen proteins that are secreted into host environments containing many plant proteases are rich in cysteine residues. These cysteine residues often form disulfide bonds that play a critical role in folding or stability of the protein [27],[28]. SnTox3 contains six cysteine residues which are predicted to form three disulfide bonds and the loss of SnTox3 activity after reduction of the protein by DTT treatment indicates that the formation of at least one disulfide bond is critical for its biological function.

*SnTox3* is expressed at high levels especially early in infection as well as in culture. The *in planta* transcription profile is similar to that of *ToxA* [15]. It is consistent with a model whereby the role of SnTox3 is to induce necrosis in susceptible host cells ahead of tissue colonization. Once the host tissue becomes necrotic, it is appropriate that *SnTox3* expression is reduced as the fungus prepares to disseminate via sporulation.

In the assembled *S. nodorum* genome, *SnTox3* is located on the end of supercontig 14 (∼1.2 Mb), however it is not clear whether this supercontig constitutes an entire chromosome. Although *SnToxA* lies in the middle of a very short supercontig (supercontig 55, ∼32 Kb), CHEF gel analysis showed it to be in a 2.35 Mb chromosome [15]. Using Southern analysis of the same CHEF blot, an *SnTox3* probe hybridized to a significantly smaller band (data not shown), indicating *SnToxA* and *SnTox3* are located in different chromosomes. It is striking that both *SnTox3* and *SnToxA* are flanked by an AT-rich sequence that contains long terminal repeat (LTR) retrotransposons [11]. We note that many other fungal avirulence and HST genes are surrounded by repetitive DNA. *AvrLm1* and *AvrLm6* conferring avirulence in *Leptosphaeria maculans* were also found residing in a genomic region rich in LTR transposons [29],[30]. Similarly, avirulence genes in *Fusarium oxysporum* f.sp. *lycopersici*, were surrounded by repetitive elements [31],[32], and *Magnaporthe grisea* avirulence genes are also found near repetitive elements and telomeres [33]. The significance of the presence of these genes in repetitive regions has yet to be determined, but it does suggest another means to identify candidate genes from sequenced fungal genomes.

Recent research in the area of pathogen effectors and host recognition has led to the theory that two layers of plant resistance are present. In the initial layer of plant defense, pathogen or microbe associated molecular patterns (PAMPs or MAMPs) are recognized by pattern recognition receptors (PRRs) at the plant cell surface, leading to PAMP triggered immunity (PTI) [26]. Pathogens can produce effectors to elude or inhibit PTI and these effectors can then be recognized in a second layer of host defense known as effector triggered immunity [26]. In this second layer of defense, the plant receptors are typically nucleotide binding-leucine rich repeat (NB-LRR) proteins which trigger resistance after direct or indirect recognition of the corresponding pathogen produced effector. Interestingly, two recent discoveries of host genes involved in HST susceptibility have been shown to be in the NB-LRR family [34],[35].

The *S. nodorum*-wheat interaction is a necrotrophic pathogen system where, like many necrotrophic systems, the pathogen induces cell death via HSTs and then thrives on dying tissue, leading to sporulation of the pathogen. Based on what is known about the ToxA-*Tsn1* model, Ptr ToxA and presumably SnToxA is recognized by the host within a few hours of infiltration [36] and internalized into the cell [17] followed by a cascade of events involving host transcription and translation [37],[38] leading to a susceptible response. Evidence suggests that *Tsn1*, the wheat gene associated with ToxA sensitivity in the *S. nodorum* and *P. tritici-repentis* systems, is also a member of the NB-LRR family (Faris et al. unpublished data) implicating another resistance-like gene in effector triggered susceptibility by a necrotrophic fungus.

In the *S. nodorum*-wheat interaction, evidence has been shown for a complex of at least four pathogen-produced effector proteins each with a distinct corresponding dominant host gene that confers susceptibility [14], one of which is likely a member of the NB-LRR gene family. The *S. nodorum*-wheat interaction is the inverse of the biotrophic host pathogen model of effector triggered immunity, in that cell death is favorable to pathogen virulence rather than to host resistance. The current work adds strength to the hypothesis that disease in the SNB system is a result of multiple effector proteins which, when produced by the pathogen, lead to effector triggered susceptibility (ETS) rather than effector triggered immunity (ETI), but possibly via pathways involving resistance-like genes. This is likely the case in other necrotrophic systems involving both proteinaceous and non proteinaceous HSTs.

A screen of a global sample of *S. nodorum* field populations revealed that 39.9% of tested strains lacked *SnTox3* and there was considerable variation in the observed frequency of *SnTox3* among geographical regions. Very similar findings emerged from an analysis of *SnToxA* using the same isolates, though *SnToxA* was deleted in almost two thirds of the tested isolates [39]. Data for both toxins were obtained from 849 of the field isolates; 164 isolates tested positive for both toxins, while 293 isolates had neither toxin. The latter finding suggests that these two toxins are not required for pathogenicity and that additional toxins or other factors are likely to be active in modern field populations of *S. nodorum*. The remaining isolates carried only one out of the two toxins discussed above. For *SnToxA* we hypothesized that the frequency of the gene in *S. nodorum* populations reflected the frequency of the *Tsn1* sensitivity gene in the corresponding wheat populations [15],[39]. Some evidence to support this hypothesis came from recent work in Australia where all of the tested *S. nodorum* isolates carried *ToxA*. Oliver et al. [40] showed that more than 90% of modern Australian wheat cultivars carry the corresponding *Tsn1* sensitivity allele. We propose the same mechanism to explain the observed differences in *SnTox3* frequencies among regional populations, with frequencies of *SnTox3* reflected by the frequencies of *Snn3* in the corresponding wheat populations. Sequence analysis of *SnTox3* loci revealed diversity at both the nucleotide and amino acid level that was similar to that observed for *SnToxA* [39]. *SnTox3* was sequenced from 245 isolates. Two of the protein variants comprised approximately 97% of the isolates, with the other two variants being rare. All four proteins exhibited full biological activity, and thus virulence, on wheat varieties carrying *Snn3*.

The availability of SnTox3 will allow wheat geneticists and breeders to use SnTox3 to identify and eliminate *Snn3* in germplasm. *SnTox3* is only the second gene cloned from the SNB system that encodes a proteinaceous necrosis inducing toxin and therefore can be used in conjunction with *SnToxA* to continue to evaluate the mechanism by which *S. nodorum* infects wheat. This susceptibility inducing effector protein is one of only a handful of proteinaceous HSTs identified and therefore is a valuable addition to the expanding body of knowledge specific to necrotrophic plant pathogen interactions, especially in the important Dothideomycete class. On a broader level, the cloning of *SnTox3* provides an additional tool to investigate how necrotrophic fungi benefit from susceptibility-inducing effector proteins.

*S. nodorum* is a member of the Dothideomycete class of fungi which is a newly classified and large fungal taxon including many important necrotrophic pathogens [41]. In addition to *S. nodorum* and *P. tritici-repentis*, proteinaceous HSTs have recently been reported from other Dothideomycete species [6]–[8]. It is possible that multiple interactions similar to the *S. nodorum*-wheat interaction are also present in other Dothideomycete disease systems. As the first Dothideomycete species to be sequenced, *S. nodorum* may serve as a model to study the necrotrophic pathogen lifestyle including the interaction with its host.

## Materials and Methods

### Plant materials

A population of recombinant inbred (RI) lines derived from a cross between the Brazilian hard red spring wheat (HRSW) breeding line BR34 and the North Dakota HRSW variety ‘Grandin’ was developed and provided by Dr. James A. Anderson, University of Minnesota. This population, consisting of 118 F_7∶9_ lines, segregates for both toxin and disease reaction to *S. nodorum*. Parental line BR34 is toxin insensitive and disease resistant and Grandin is toxin sensitive and disease susceptible. Several BG RI lines have been selected as toxin differential lines including BG261 (SnToxA sensitive only, [15]), BG223 (SnTox2 sensitive only, [19]) and BG220 (SnTox3 sensitive only, [20]). The entire population, with parental lines and selected toxin differential lines were used in this study for testing toxin production and/or virulence of different genetically modified *P. pastoris* and *S. nodorum* fungal strains along with wild type strains or isolates. All plants were grown in plastic cones containing SB100 professional grow mix (Sungrow Horticulture, Dellevue, WA) in the greenhouse at an average temperature of 21°C with a 14-h photoperiod.

### Toxin bioassay and fungal inoculation

Toxin bioassay and fungal inoculation were conducted at the two- to three-leaf stage following the procedures previously described [15]. For toxin bioassays, the necrotic reaction was recorded as either sensitive or insensitive based on the presence or absence of necrosis in the infiltrated area. For fungal inoculation, disease ratings followed the 0–5 disease scale developed by Liu et al. [21] with 0 being highly resistant and 5 highly susceptible. Two replicates of at least three plants of each line were evaluated for toxin sensitivity and three replicates of three plants of each line were evaluated for fungal inoculation. An overall average from the three replicates was calculated and used in statistical analysis.

### Purification of SnTox3

SnTox3 was partially purified and characterized as described in Friesen et al. [20] using *S. nodorum* isolate Sn1501. Recently the highly virulent North Dakota *S. nodorum* isolate Sn4, was identified as a better toxin producer, therefore this isolate was used for production of SnTox3. Culture filtrate production and partial purification was done as described by Friesen et al. [20] with some modifications. Briefly, initial purification steps were conducted using the ÄKTA prime plus (GE Healthcare, Piscataway, NJ) liquid chromatography system. In order to obtain enough protein for mass spectrometry, ∼300 ml of 3 week old fungal culture filtrate was dialyzed overnight against a water using a 3.5 kDa molecular weight cutoff dialysis tubing (Fisher Scientific, Pittsburgh, PA). The dialyzed culture filtrate was loaded onto a HiPrep SPXL 16/10 cation exchange column (GE Healthcare Piscataway, NJ) after pre-equilibration with the same buffer used in dialysis. After loading the sample, the column was washed with 50 ml of 20 mM sodium acetate buffer pH 5.0 followed by a gradient elution of 0–300 mM sodium chloride plus 20 mM sodium acetate pH 5.0 at a flow rate of 5.0 ml/min over 20 min. The 5 ml fractions were collected and tested on the SnTox3 differential line BG220. The most active fraction was used for size exclusion chromatography using a HiLoad 16/60 Superdex 30 prep-grade gel filtration column. The sample was injected using a 5 ml loop and 20 mM sodium acetate, 50 mM NaCl, pH 5.0 running buffer was used with a flow rate of 1 ml/min and a fraction size of 5 ml. The size-based fraction containing SnTox3 was further separated using SDS-PAGE gel electrophoresis. The sample was loaded into a precast 16.5% tris-tricine polyacrylamide gel (Bio-Rad, Hercules, CA) and subjected to electrophoresis in a Bio-Rad Mini PROTEAN 3 system (Bio-Rad, Hercules, CA) using the buffer system of Shägger and von Jagow [42]. The gel was stained with Coomassie Blue solution (0.2% Coomassie Blue R250, 7.5% acetic acid and 50% ethanol) to visualize the protein bands. Based on a pre-stained protein standard (Bio-Rad, Hercules, CA), the bands within the expected size range (10–30 kDa) were excised individually from the gel and subjected to mass spectrometric analysis.

### Mass spectrometry and identification of the SnTox3-encoding gene

Matrix-assisted laser desorption/ionization mass spectrometry with automated tandem time of flight fragmentation of selected ions (MALDI-TOF/TOF) of trypsin digested proteins were acquired with a 4700 Proteomics Analyzer mass spectrometer (Applied Biosystems, Framingham, MA) in the positive reflectron mode. Spectra were obtained by averaging 1000 and 2500 acquired spectra in the MS and MS/MS mode, respectively. Post source decay fragmentation MS/MS spectra of selective peptides were obtained with 1 keV acceleration voltage. Conversion of TOF to mass (Da) for the monoisotopic ions, [M+H]^+^, was based on calibration of the instrument with a peptide standard calibration kit (Applied Biosystems). The MS and MS/MS spectra were combined and searched against the sequence of the *S. nodorum* protein database (http://www.broad.mit.edu/annotation/genome/stagonospora_nodorum) using the Mascot (Matrix Science, Inc. Boston, MA) search engine through GPS Explorer Software (Applied Biosystems) with a 50 ppm and 0.1 Da error tolerance for MS and MS/MS spectra, respectively, one missed trypsin cleavage allowance, oxidation of methionine, and carbamidomethyl derivatization of reduced cysteine as a variable modification. The signal to noise ratio for peak filtering was set to 10 for MS and 20 for MS/MS. The threshold for proteins from database searches (MS+MS/MS and MS/MS) was set within a ≥95% confidence interval (*P*≥0.05). Protein bands likely containing SnTox3 were subjected to digestion using Trypsin Gold, mass spectrometry grade (Promega Co., Madison, WI), following manufacture procedures and ZipTips protocols for sample cleaning and spotting in a MALDI plate. Alpha-cyano-4-hydroxycinamic acid was used as a matrix for mass spectrometry analysis. Using the *S. nodorum* genome sequence (http://www.broad.mit.edu/annotation/genome/stagonospora_nodorum), each identified protein was used to identify the genomic DNA sequence for the encoding gene, along with its 5′ and 3′ flanking region. Primers for identified genes (*SNOG_08981* and *SNOG_16063*): 8981g1F with 8981g1R, and 16063g1F with 16063g1R (Table S1) were designed using the web-based program Primer3 [43] (http://frodo.wi.mit.edu/). PCR was performed with an annealing temperature of 60°C to verify the presence or absence of each gene in both virulent and avirulent isolates. Candidate proteins were subjected to further validation if they were found to be absent in the avirulent isolate Sn79-1087 and present in both SN15 and Sn4.

### Fungal isolates and Southern analysis of the occurrence of the SnTox3-encoding gene

Nine *S. nodorum* isolates, collected from different locations, were used to investigate the occurrence of the SnTox3-producing gene. The origin and toxin production of those isolates are listed in Table 1. Three related fungal species isolates were also included in this study including: *P. teres* Pt-15A (obtained from B. Steffenson Univ. of MN St. Paul, MN USA), *P. tritici-repentis* Ptr86-124, (obtained from L. Lamari Univ. of Manitoba, Winnipeg, Canada), and *Mycosphaerella graminicola* Str975, (obtained from C. Hollingsworth, Univ. of MN Crookston, MN USA). Fungal DNA extractions were carried out as previously described [44],[45]. A total of 5 µg of genomic DNA of each isolate was completely digested with the restriction enzyme *Eco*RI at 37°C. DNA blotting, Southern probe preparation, hybridization, and signal detection were performed as described by Faris et al [46]. The full length *SnTox3* gene, a 693 bp fragment, was PCR amplified from SN15 with primer pair 8981cF and 8981cR (see Table S1 for sequence) and used as a probe.

### *SnTox3* full length cDNA amplification and 5′ and 3′ RACE

Total RNA was prepared from Sn4 using TRIzol Reagents (Invitrogen, Carlsbad, CA) according to the manufacturer's protocol and treated with RNase-free DNase I (Promega Madison, WI). The one step RT PCR kit (Invitrogen Carlsbad, CA) containing reverse-transcriptase and a DNA *Taq* polymerase mix was used to amplify a full length cDNA of *SnTox3* using primers 8981cF and 8981cR (Table S1). To make sure there was no DNA contamination, one control PCR reaction was set up with only *Taq* polymerase (New England Biolabs Ipswich, MA). The amplified fragment was excised from the agarose gel, gel-purified and cloned into the TopoTA vector (Invitrogen, Carlsbad CA). To obtain the 5′ and 3′ end of the cDNA, 5′ and 3′ RACE were performed using the Smart RACE cDNA amplification kit (Stratagene, La Jolla, CA) according to the instructions in the user's manual. The amplified 5′ and 3′ RACE fragments were gel-purified and cloned into the TopoTA vector. All of the plasmids were prepared with the plasmid DNA miniprep kit (Qiagen, Valencia, CA) and sequenced from both directions with M13 forward and reverse primers. The sequences were then used to assemble the full length cDNA, including the 5′ and 3′ untranslated region (UTR) based on the genomic sequence.

The search of DNA sequence with similarity to *SNOG_08981* cDNA was performed using BLASTN [47] and BLASTX [48] against the public NCBI non-redundant (NR) database (http://blast.ncbi.nlm.nih.gov/Blast.cgi) and the annotated *S. nodorum* database at the Broad institute website (http://www.broad.mit.edu/annotation/genome/stagonospora_nodorum.2/Blast.html).

### RNA isolation and quantitative real time Q-PCR

To investigate the transcriptional expression pattern of *SNOG_08981* during infection, SN15 conidia were inoculated onto the susceptible wheat variety ‘Amery’ and total RNA was isolated from inoculated leaves at 3, 5, 7 and 10 days after inoculation. RNA isolation, cDNA synthesis and gene transcript abundance analysis were performed as previously described [49]. Intron-spanning primers ActinF and ActinR (Table S1) designed to amplify actin (*Act1*; Genbank accession number EAT90788) were used to check all cDNA samples [50] and were shown to be free of genomic DNA (data not shown). *SnTox3* expression was examined using Tox3qPCRF and Tox3qPCRR primers (Table S1). *Act1* was used as a constitutively expressed control for the normalisation of *SnTox3* expression using ActinqPCRf and ActinqPCRr primers (Table S1). All reactions were performed in technical duplicates from pooled biological triplicates.

### Heterologous expression of *SnTox3* in *Pichia pastoris*

The yeast strain *Pichia pastoris* X33 and vector pGAPZA for gene expression were provided in the commercial kit developed for constitutive expression and purification of recombinant proteins (Invitrogen, Carlsbad CA). The pGAPZA vector is ∼2.9 kb and contains the Zeocin resistance gene (*Sh ble*) as a selectable marker for use in both *E. coli* and yeast. The full length of *SnTox3* cDNA in TopoTA was re-amplified with primer pairs 8981cF_*Eco*RI and 8981cR_ApaI (see Table S1) which contain *Eco*RI and *Apa*I restriction sites, respectively. The re-amplified *SnTox3* cDNA with restriction sites was cloned into the TopoTA vector. After sequencing confirmation for sequence identity, the full length *SnTox3* cDNA was released from the TopoTA vector by digestion with *Eco*RI and *Apa*I and directionally cloned into the pGAPZA vector. The expression constructs were sequenced with the primer pGAPF and 3′Aox1 (see Table S1) for checking the gene identity and the translation reading frame. At least 5 µg of the construct DNA linearized with *Bsr*DI was used for yeast transformation. The preparation and transformation of competent *P. pastoris* cells were done using the *Pichia* EasyComp kit (Invitrogen, Carlsbad, CA) following the steps described in the user manual.

Four different positive *Pichia* clones were selected from the transformation plate to test toxin expression. Each clone was picked and cultured in 1 ml YPD liquid medium (1% yeast extract, 2% peptone and 2% dextrose) in 15 ml tubes. Since SnTox3 are expected to be secreted, *P. pastoris* cells were centrifuged at 6000×g for 5 min and the supernatant was used for testing toxin activity.

### Transformation and disruption of the *SnTox3* gene

#### SnTox3 expression vector construction

The previously published vector, pDAN [15], containing a cpc-1::hygromycin-resistance gene cassette was used to carry the *SnTox3* gene for transformation into the avirulent isolate Sn79-1087. The primer pair 8981g1F_XbaI and 8981g1R_XbaI (Table S1) containing an *Xba*I restriction site was used to amplify a ∼1.2 kb genomic region (Figure 2B) of *SnTox3* which contained a putative *SnTox3* promoter region and terminator. This ∼1.2 kb fragment was first cloned into the TopoTA vector to form the plasmid pTopoSnTox3, followed by sequence confirmation of the *Xba*I restriction site and *SnTox3* sequence using M13 forward and reverse primers. The remaining steps to put SnTox3 into the pDAN vector followed the same procedure as that used for SnToxA [15]. The resulting plasmid was screened and verified using the primer pairs 8981g1F_XbaI and 8981g1R_XbaI (Table S1).

#### SnTox3 disruption vector construction

*SnTox3* has a unique *Sal*I restriction site located near the middle of the ORF that provides a site for the insertion of the hygromycin-resistance gene to form the gene disruption vector. The plasmid pTopoSnTox3 was cut open via *Sal*I digestion and re-ligated with the hygromycin resistance gene (*HYG*) cassette which had been released from the pLP605KO vector [51] using the restriction enzyme *Xho*I. The recombinant DNA was transformed into DH5α *E. coli* competent cells (Invitrogen, Carlsbad, CA) and screened with PCR using the primer pair 8981g1F_XbaI and HY (Table S1). In the recombinant vector, the *SnTox3* fragment was therefore separated by the *HYG* gene cassette as ∼700 bp of 5′ region and ∼500 bp of 3′ region (Figure 6B) for homologous recombination. The whole construct was released from the TopoTA backbone as a ∼3.8 kb fragment using *Xba*I before transformation into Sn1501.

#### Fungal protoplasting and transformation

The fungal protoplasting and PEG-mediated transformation methods described by Solomon et al. [45] were used to introduce the expression vector into the avirulent isolate Sn79-1087 and the disruption vector into the virulent isolate Sn1501. The regenerated colonies were screened for gene integration using the corresponding primer pair (8981g1F and 8981g1R for *SnTox3* transformation in Sn79-1087, 8981g0F and HY for *SnTox3* disruption in Sn1501). RT-PCR with primers 8981cF and 8981cR and Southern analysis with full length *SnTox3* was performed on the selected transformants to validate *SnTox3* transformation and disruption. The *S. nodorum* actin gene primer [50] was used to amplify the actin gene as an internal control in RT-PCR.

### Molecular mapping and QTL analysis

A wheat genetic map containing 787 DNA markers has been developed in the BG population [52],[53]. The marker density of the maps makes this population ideal for mapping genes and doing QTL analysis of the *S. nodorum*-wheat interaction. In this population, sensitivity to SnToxA, SnTox2 and SnTox3 have been mapped to wheat chromosome arms 5BL [15],[22], 2DS [19] and 5BS [20], respectively. To verify that SnTox3 has been produced by genetically modified yeast strains and *S. nodorum* isolates the culture filtrate or partially purified toxin prep from the transformed isolates were infiltrated on the 118 recombinant inbred lines to map the sensitivity loci and verify that they correlate with the previously mapped toxin sensitivity loci. One Sn79-1087 *SnTox3* transformant and two Sn1501 *SnTox3* knockout transformants were also inoculated onto this population using wild type and ectopic transformants as controls. The molecular mapping and QTL analysis in the BG population was performed as previously described [20].

### Prediction of signal peptides and disulfide bonds in SnTox3 protein

Two web-based programs SignalP 3.0 (http://www.cbs.dtu.dk/services/SignalP/) and WolfPsort (http://wolfpsort.org/) were used to predict the cellular location of SnTox3 and determine the presence and length of a signal peptide. The protein sequence was submitted to the website PredictProtein to run DISULFIND [54] to identify the disulfide bond prediction. To investigate if the predicted disulfide bonds were important for toxin activity, the *P. pastoris* culture containing SnTox3 was treated with Dithiothreitol (DTT) (Fisher Scientific, Pittsburgh, PA) at two different concentrations including 5 mM and 10 mM, with water added as a negative control. The treated culture filtrates were assayed on line BG220 along with an untreated negative control.

### Genetic diversity analysis

PCR amplification was used to screen 923 isolates for the presence or absence of *SnTox3*. PCR primer pairs, 8981cF-R and 8981g1F-R were both used to confirm *SnTox3* presence. The annealing temperature of all PCRs was 55°C. Sequencing of the PCR products was performed with the same primers using an ABI 3730 automated sequencer (Applied Biosystems, Foster City, CA). Alignment of forward and reverse sequences for each isolate was performed in SeqScape software V2.5 (Applied Biosystems, Foster City, CA). The same software was used for translation and identification of protein haplotypes. Gene diversity (Pi) was measured using the nucleotide alignment software DNAsp [55].

*S. nodorum* isolates were obtained from eight major geographical regions; North America (358), Central America (41), Europe (192), Iran (47), Australia (57), Central Asia (49), East Asia (107), and South Africa (74). The location, year of collection and frequency of *SnTox3* deletions is summarized for each population in Table S2. Each of these populations was characterized previously for *SnToxA* [39]. Isolation procedures and DNA extractions were performed according to the procedures described previously [56]. A total of 250 ng of genomic DNA was spotted as per manufacturer instructions using the BioDot Microfiltration Apparatus (BioRAD, Hercules, CA). Hybridization and signal detection was performed as previously described [46].

The other three protein variants of SnTox3 were amplified from a representative isolate and transformed into *P. pastoris* following the same procedure described earlier for SnTox3 in isolate Sn4. In order to test the effectiveness of each toxin variant, culture filtrates were produced and assayed on the SnTox3 differential line BG220.

## Supporting Information

Table S1 Primers used in this study.
(0.04 MB DOC)

Table S2 *S. nodorum* populations used in the investigation of genetic diversity of *SnTox3*.
(0.56 MB DOC)

Figure S1 Partial purification of SnTox3 by ion exchange (A) and size exclusion (B) chromatography. PrimeView software (GE healthcare, Piscataway, NJ) was used to create chromatogram graphs with blue and green showing the instant UV absorbance, and % buffer B (300 mM NaCl), respectively. A. Ion exchange with a 0 to 300 mM NaCl gradient elution of Sn4 dialyzed culture filtrates. The Y-axis indicates the UV absorbance (blue line) and X-axis indicates the fraction number. The fractions containing SnTox3 activity are indicated by 3 plus (high activity), 2 plus (moderate activity) and 1 plus (low activity). B. Size exclusion chromatography of fraction 17 from the previous ion exchange chromatography. The Y-axis indicates the UV absorbance (blue line) and the X-axis indicates the fraction number. The fractions containing SnTox3 activity were indicated by 2 plus (moderate activity) and 1 plus (low activity).
(0.02 MB TIF)

Figure S2 PCR testing of *SNOG_08981* and *SNOG_16063* identified from mass spectrometry for presence in Sn79-1087. The genomic region of two genes were amplified from Sn15 (Lane 1 and 5), Sn4 (Lane 2 and 6), Sn79-1087(Lane 3 and 7) and water controls (lane 4 and 8) using primer pairs 8981g1F and 8981g1R for *SNOG_08981*, 16063g1F and 16063g1R for *SNOG_16063* (See Table S1 for primer sequences). Hyperladder I from Bioline was used as a size standard. The fragment size from SN15 was 1,186 bp and 824 bp for *SNOG_08981* and *SNOG_16063*, respectively.
(2.55 MB TIF)

Figure S3 Protein view of SNOG_08981 from the mascot search report. A. Summary of SNOG_08981 in the mascot search. B. The deduced amino acid sequence of SNOG_08981. Red peptides were revealed by the mascot search. C. A list of SNOG_08981 peptide hits identified by the mascot search.
(0.89 MB TIF)

Figure S4 Toxin bioassay of *P. pastoris* transformed with an empty express vector. The leaves of BR34 (a), Grandin (b), BG261 (c), BG220 (d), and BG223 (e) were infiltrated with culture filtrates from a yeast strain transformed with an empty expression vector and photographed 3 days after infiltration.
(3.82 MB TIF)

Figure S5 Virulence analysis of Sn1501 and its *SnTox3* disrupted strains as well as an ectopic transformant on BR34, Grandin and the SnToxA and SnTox2 toxin differential lines. A. Parental line BR34 inoculated with Sn1501 wild type (a), Sn1501ΔSnTox3A (b) Sn1501ΔSnTox3B (c) and Sn1501Ect (d). All four strains show avirulence. B. Parental line Grandin inoculated with Sn1501 wild type (a), Sn1501ΔSnTox3A(b) Sn1501ΔSnTox3B(c) and Sn1501Ect(d); wild type and ectopic type strains are more virulent than the two SnTox3-disrupted strains. C. BG261 differential line (sensitive to SnToxA only) inoculated with Sn1501 wild type (a), Sn1501ΔSnTox3A(b) Sn1501ΔSnTox3B(c) and Sn1501Ect(d); all four strains are avirulent due to the absence of *SnToxA* in all strains. D. BG223 differential line (sensitive to SnTox2 only) inoculated with Sn1501 wild type (a), Sn1501ΔSnTox3A(b) Sn1501ΔSnTox3B(c) and Sn1501Ect(d); all four strains are equally virulent due to the presence of *Snn2*.
(9.53 MB TIF)

## References

[1] Chisholm ST, Coaker G, Day B, Staskawicz BJ (2006). Host-microbe interactions: Shaping the evolution of the plant immune response.. Cell.

[2] Wolpert TJ, Dunkle LD, Ciuffetti LM (2002). Host-selective toxins and avirulence determinants: what's in a name?.. Annu Rev Phytopathol.

[3] Panaccione DG, Johnson RD, Rasmussen JB, Friesen TL, Kempken F (2002). Fungal phytotoxins.. The Mycota XI Agricultural Applications.

[4] Ciuffetti LM, Tuori RP, Gaventa JM (1997). A single gene encodes a selective toxin causal to the development of tan spot of wheat.. Plant Cell.

[5] Martinez JP, Ottum SA, Ali S, Francl LJ, Ciuffetti LM (2001). Characterization of the *ToxB* gene from *Pyrenophora tritici-repentis*.. Mol Plant Microbe In.

[6] Barthe P, Pujade-Renaud V, Breton F, Gargani D, Thai R (2007). Structure analysis of cassiicolin a host-selective protein toxin from *Corynespora cassiicola*.. J Mol Biol.

[7] Parada RY, Sakuno E, Mori N, Oka K, Egusa M (2008). *Alternaria brassicae* produces a host-specific protein toxin from germinating spores on host leaves.. Phytopathology.

[8] Sarpeleh A, Wallwork H, Tate ME, Catcheside DEA, Able AJ (2008). Initial characterization of phytotoxic proteins isolated from *Pyrenophora teres*.. Physiol and Mol Plant Pathol.

[9] Eyal Z (1981). Integrated control of Septoria disease of wheat.. Plant Dis.

[10] Solomon PS, Lowe RGT, Tan K-C, Waters ODC, Oliver RP (2006). *Stagonospora nodorum*: cause of stagonospora nodorum blotch of wheat.. Mol Plant Pathol.

[11] Hane JK, Lowe RGT, Solomon PS, Tan K-C, Schoch CL (2007). Dothideomycete-plant interactions illuminated by genome sequencing and EST analysis of the wheat pathogen *Stagonospora nodorum*.. Plant Cell.

[12] Eyal Z, Lucas JA, Bowyer P, Anderson HM (1999). Breeding for resistance to *Septoria* and *Stagonospora* disease of wheat.. *Septoria* on Cereals—A Study of Pathosystems.

[13] Xu SS, Friesen TL, Cai X (2004). Sources and Genetic Control of Resistance to Stagonospora Nodorum Blotch in Wheat.. In: Research Signpost/recent Research Development in Gentics and Plant Breeding, Book Chapter Vol.

[14] Friesen TL, Faris JD, Solomon PS, Oliver RP (2008). Host-specific toxins: effectors of necrotrophic pathogenicity.. Cell Microbiol.

[15] Friesen TL, Stukenbrock EH, Liu ZH, Meinhardt SW, Ling H (2006). Emergence of a new disease as a result of interspecific virulence gene transfer.. Nat Genet.

[16] Faris JD, Anderson JA, Francl LJ, Jordahl JG (1996). Chromosomal location of a gene conditioning insensitivity in wheat to a necrosis-inducing culture filtrate from *Pyrenophora tritici-repentis*.. Phytopathology.

[17] Manning VA, Ciuffetti LM (2005). Localization of Ptr ToxA produced by *Pyrenophora tritici-repentis* reveals protein import into wheat mesophyll cells.. Plant Cell.

[18] Liu ZH, Faris JD, Meinhardt SW, Ali S, Rasmussen JB (2004). Genetic and physical mapping of a gene conditioning sensitivity in wheat to a partially purified host selective toxin produced by *Stagonospora nodorum*.. Phytopathology.

[19] Friesen TL, Meinhardt SW, Faris JD (2007). The *Stagonospora nodorum*–wheat pathosystem involves multiple proteinaceous host selective toxins and corresponding host sensitivity genes that interact in an inverse gene-for-gene manner.. Plant J.

[20] Friesen TL, Zhang Z, Solomon PS, Oliver RP, Faris JD (2008). Characterization of the interaction of a novel *Stagonospora nodorum* host-selective toxin with a wheat susceptibility gene.. Plant Physiol.

[21] Liu ZH, Friesen TL, Meinhardt SW, Ali S, Rasmussen JB (2004). Quantitative trait loci analysis and mapping of seedling resistance to *Stagonospora nodorum* leaf blotch in wheat.. Phytopathology.

[22] Liu ZH, Friesen TL, Ling H, Meinhardt SW, Oliver RP (2006). The *Tsn1*-ToxA interaction in the wheat-*Stagonospora nodorum* pathosystem parallels that of the wheat-tan spot system.. Genome.

[23] Nei M (1987). Molecular Evolutionary Genetics.

[24] Solomon PS, Wilson TJ, Rybak K, Parker K, Lowe RGT (2006). Structural characterisation of the interaction between *Triticum aestivum* and the dothideomycete pathogen *Stagonospora nodorum*.. Eur J Plant Pathol.

[25] Oliver RP (2009). Plant breeding for disease resistance in the age of effectors.. Phytoparasitica.

[26] Boller T, Felix G (2009). A renaissance of elicitors: perception of microbe-associated molecular patterns danger signals by pattern recognition receptors.. Annu Rev Plant Biol.

[27] Rep M (2005). Small proteins of plant-pathogenic fungi secreted during host colonization.. FEMS Microbiol Lett.

[28] Luderer R, de Kock MJD, Dees RHL, de Wit PJGM, Joosten MHAJ (2002). Functional analysis of cysteine residues of ECP elicitor proteins of the fungal tomato pathogen *Cladosporium fulvum*.. Mol Plant Pathol.

[29] Gout L, Fudal I, Kuhn ML, Blaise F, Eckert M (2006). Lost in the middle of nowhere: The *AvrLm1* avirulence gene of the Dothideomycete *Leptosphaeria maculans*.. Mol Microbiol.

[30] Fudal I, Ross S, Gout L, Blaise F, Kuhn ML (2007). Heterochromatin-like regions as ecological niches for avirulence genes in the *Leptosphaeria maculans* genome: Map-based cloning of AvrLm6.. Mol Plant Microbe In.

[31] Rep M, van der Does HC, Meijer M, van Wijk R, Houterman PM (2004). A small, cysteine-rich protein secreted by *Fusarium oxysporum* during colonization of xylem vessels is required for *I-3*-mediated resistance in tomato.. Mol Microbiol.

[32] Houterman PM, Cornelissen BJC, Rep M (2008). Suppression of Plant Resistance Gene-Based Immunity by a Fungal Effector.. PLoS Pathog.

[33] Zhou E, Jia Y, Singh P, Correll JC, Lee FN (2007). Instability of the *Magnaporthe oryzae* avirulence gene *AVR-Pita* alters virulence.. Fungal Genet Biol.

[34] Lorang JM, Sweat TA, Wolpert TJ (2007). Plant disease susceptibility conferred by a “resistance” gene.. P Natl Acad Sci USA.

[35] Nagy ED, Bennetzen JL (2008). Pathogen corruption and site-directed recombination at a plant disease resistance gene cluster.. Genome Res.

[36] Kwon CY, Rasmussen JB, Francl LJ, Meinhardt SW (1996). A quantitative bioassay for necrosis toxin from *Pyrenophora tritici-repentis* based on electrolyte leakage.. Phytopathology.

[37] Kwon CY, Rasmussen JB, Meinhardt SW (1998). Activity of Ptr ToxA from *Pyrenophora tritici-repentis* requires host metabolism.. Physiol Mol Plant P.

[38] Rasmussen JB, Kwon CY, Meinhardt SW (2004). Requirement of host signaling mechanisms for the action of Ptr ToxA in wheat.. Eur J Plant Pathol.

[39] Stukenbrock EH, McDonald BA (2007). Geographical variation and positive diversifying selection in the host-specific toxin SnToxA.. Mol Plant Pathol.

[40] Oliver RP, Lord A, Rybak K, Faris JD, Solomon PS (2008). Emergence of tan spot disease caused by toxigenic *Pyrenophora tritici-repentis* in Australia is not associated with increased deployment of toxin-sensitive cultivars.. Phytopathology.

[41] Schoch CL, Shoemaker RA, Seifert KA, Hambleton S, Spatafora JW (2006). A multigene phylogeny of the Dothideomycetes using four nuclear loci.. Mycologia.

[42] Schägger H, von Jagow G (1987). Tricine-sodium dodecyl sulfate-polyacrylamide gel electrophoresis for the separation of proteins in the range from 1 to 100 kDa.. Anal Biochem.

[43] Rozen N, Skaletsky H (2000). Primer3 on the WWW for general users and for biologist programmers.. Methods Mol Biol.

[44] Friesen TL, Ali S, Klein KK, Rasmussen JB (2005). Population genetic analysis of a global collection of *Pyrenophora tritici-repentis*, causal agent of tan spot of wheat.. Phytopathology.

[45] Solomon PS, Thomas SW, Spanu P, Oliver RP (2003). The utilisation of di/tripeptides by Stagonospora nodorum is dispensable for wheat infection.. Physiol Mol Plant P.

[46] Faris JD, Haen KM, Gill BS (2000). Saturation mapping of a gene rich recombination hot spot region in wheat.. Genetics.

[47] Zhang Z, Schwartz S, Wagner L, Miller W (2000). A greedy algorithm for aligning DNA sequences.. J Comput Biol.

[48] Altschul SF, Madden TL, Schäffer AA, Zhang J, Zhang Z (1997). “Gapped BLAST and PSI-BLAST: a new generation of protein database search programs”.. Nucleic Acids Res.

[49] Solomon PS, Tan K-C, Oliver RP (2003). The nutrient supply of pathogenic fungi; a fertile field for study.. Mol Plant Pathol.

[50] Tan K-C, Heazlewood JL, Millar AH, Thomson G, Oliver RP (2008). A signaling-regulated short-chain dehydrogenase of *Stagonospora nodorum* regulates asexual development.. Eukaryot Cell.

[51] Panaccione DG, Johnson RD, Wang J, Young CA, Damrongkool P (2001). Elimination of ergovaline from a grass-*Neotyphodium* endophyte symbiosis by genetic modification of the endophyte.. P Natl Acad Sci USA.

[52] Liu ZH, Anderson JA, Hu J, Friesen TL, Rasmussen JB (2005). A wheat intervarietal genetic linkage map based on microsatellite and target region amplified polymorphism markers and its utility for detecting quantitative trait loci.. Theor Appl Genet.

[53] Zhang Z, Friesen TL, Simons KJ, Xu SS, Faris JD (2008). Identification development and validation of markers for marker-assisted selection against the *Stagonospora nodorum* toxin sensitivity genes *Tsn1* and *Snn2* in wheat.. Mol Breeding.

[54] Vullo A, Frasconi P (2004). Disulfide connectivity prediction using recursive neural networks and evolutionary information.. Bioinformatics.

[55] Rozas J, Sanchez-DelBarrio JC, Messeguer X, Rozas R (2003). DnaSP DNA polymorphism analyses by the coalescent and other methods.. Bioinformatics.

[56] McDonald BA, Miles J, Nelson LR, Pettway RE (1994). Genetic variability in nuclear DNA in field populations of *Stagonospora nodorum*.. Phytopathology.

