# Supplementary material for: SnTox3 Acts in Effector Triggered Susceptibility to Induce Disease on Wheat Carrying the Snn3 Gene
Source: PLoS Pathog. 2009 Sep 18;5(9):e1000581. doi: 10.1371/journal.ppat.1000581 (PMC2736379; doi:10.1371/journal.ppat.1000581)
Supplement: Table S1 — Primers used in this study. (0.04 MB DOC) [file ppat.1000581.s001.doc]

Supporting Table 1. Primers used in this study

| Primer pair* | Sequence^¶^ | Amplicon size (bp) | Application |
| --- | --- | --- | --- |
| 8981g1F | TTCGACAGCCTTCACACGTA | 1185 | PCR testing of the presence of *SNOG_08981* in Sn791087 |
| 8981g1R | ACAACGAGATTTGGCTTTGC |
| 16063g1F | TTGAAGGCGTTGTACGAGTG | 897 | PCR testing of the presence of *SNOG_16063* in Sn79-1087 |
| 16063g1R | TAGCACCGACAGCATCCCAGGCT |
| 8981cF | ATGCATTTTACAAAGTTCCT | 693 | Amplification of *SnTox3*  5’ and 3’ ends and full length cDNA and RT-PCR of *SnTox3* expression |
| 8981cR | CTACTCCCCTCGTGGGATTGCCCCAT |
| 8981cF_EocRI | *GAATTC*AGCTATGCATTTTACAAAGTTCCT | 708 | Amplification of *SnTox3* full length cDNA for cloning and yeast expression |
| 8981cR_ApaI | *GGGCCC*TACTCCCCTCGTGGGATTGCCCCAT |
| ActinF | CTGCTTTGAGATCCACAT | 255 | S. nodorum actin gene as internal control for RT-PCR |
| ActinR | GTCACCACTTTCAACTCC |
| 8981qPCRF | AATGTCGACCGTTTTGACC | 143 | Amplification of partial of *SnTox3* in Q-PCR analysis |
| 8981qPCRR | GGTTGCCGCAGTTGATATAA |
| ActinqPCRf | AGTCGAAGCGTGGTATCCT | 165 | Amplification of partial of *S*. *nodorum* actin gene in Q-PCR analysis |
| ActinqPCRr | ACTTGGGGTTGATGGGAG |
| 8981g1F_XbaI | *TCTAGA*TTCGACAGCCTTCACACGTA | 1197 | Amplification of genomic region for cloning and transformation into Sn79-1087 |
| 8981g1R_XbaI | *TCTAGA*ACAACGAGATTTGGCTTTGC |
| pGAPF | GTCCCTATTTCAATCAATTGAA | - | Sequencing yeast expression construct from 5’ |
| 3’AOX1 | GCAAATGGCATTCTGACATCC | - | Sequencing yeast expression construct from 3’ |
| 8981g0F | ATCCCAGACATCCCACTCAA | ~2,900^†^ | Screening of *SnTox3*-disrupted transformants in Sn1501. |
| HY | GGATGCCTCCGCTCGAAGTA |

* *Eco*RI, *Apa*I and *Xba*I restriction site was added to 5’ end of some primers for directional cloning or plasmid linearization purpose.

¶ The italic letters indicate the restriction site which was added to this primer. Sequence for S. nodorum actin gene primer (ActinF and ActinR) were obtained from Tan et al.(2008)[50]. The sequence of primer (pGAPF and 3’AOX) for sequencing *SnTox3* yeast expression construct was provided in yeast expression manual (Invitrogen, Carlsbad CA). The sequences for other primers were designed by authors using web-based program primer3.0. All the primers were designed with Tm greater than 60°C and PCR were all conducted at an annealing temperature at 60°C.

† The size for this PCR fragment was based on a close estimation.
