# Supplementary material for: SnTox3 Acts in Effector Triggered Susceptibility to Induce Disease on Wheat Carrying the Snn3 Gene
Source: PLoS Pathog. 2009 Sep 18;5(9):e1000581. doi: 10.1371/journal.ppat.1000581 (PMC2736379; doi:10.1371/journal.ppat.1000581)
Supplement: Table S2 — S. nodorum populations used in the investigation of genetic diversity of SnTox3. (0.56 MB DOC) [file ppat.1000581.s002.doc]

Supporting Table 2. *S. nodorum* populations used in the investigation of genetic diversity of *SnTox3*.


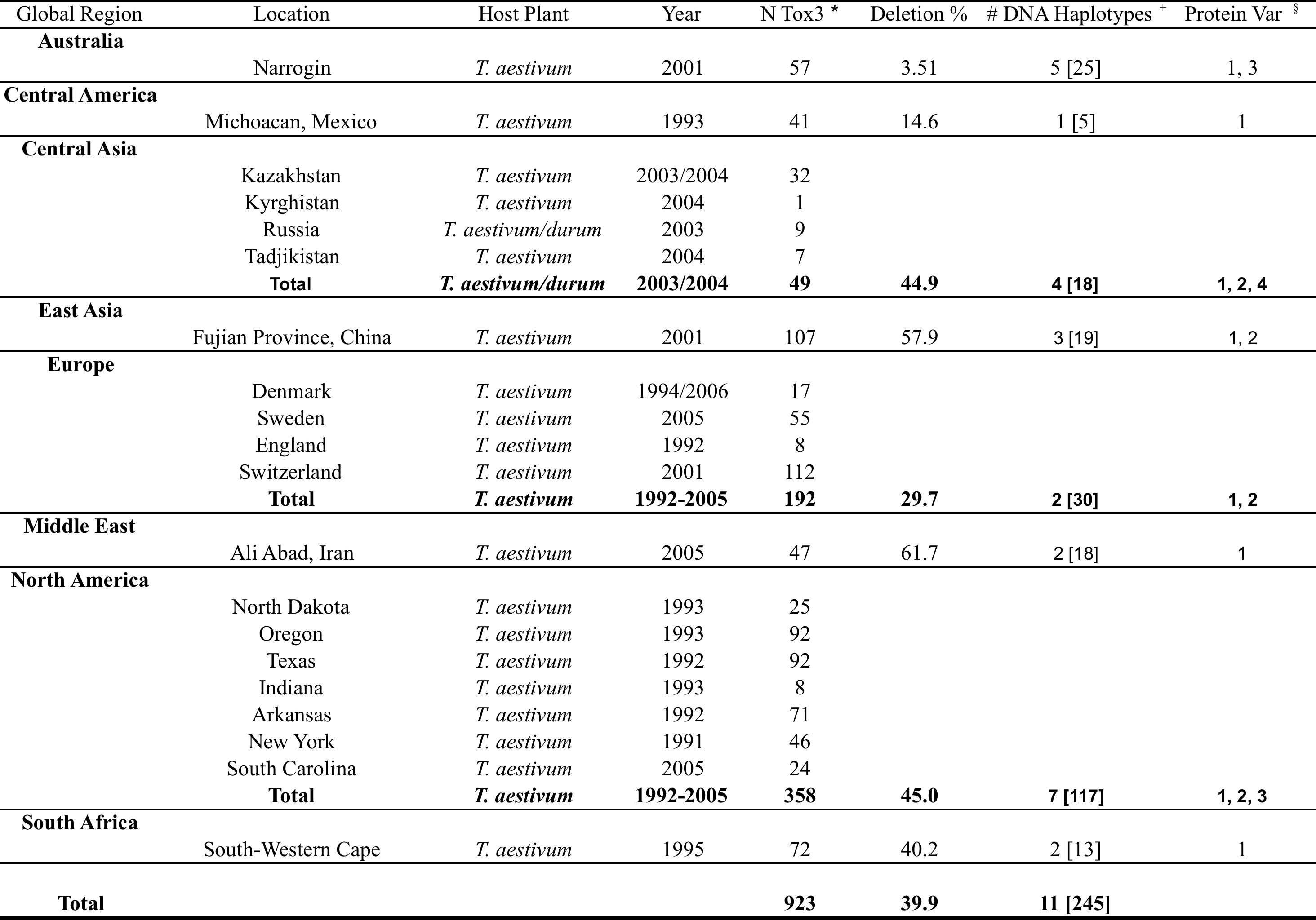


**^*^** Total number of isolates screened, using PCR, for the presence of *SNTox3* in 8 Global regions*.* Bold numbers represent totals from regions where isolates originate from several different locations.

**^+^**  In brackets, the total number of isolates that were sequenced for the *SNTox3* gene in each region. The number of DNA haplotypes found among those isolates is indicated in the preceding number. There are shared haplotypes among regions (data not shown).

**^§^** Lists the Protein Variants discovered among the DNA haplotypes. Numbers refer to Protein Variants 1-4 as discussed in this text.
