# Supplementary figures and images for: SnTox3 Acts in Effector Triggered Susceptibility to Induce Disease on Wheat Carrying the Snn3 Gene
Source: PLoS Pathog. 2009 Sep 18;5(9):e1000581. doi: 10.1371/journal.ppat.1000581 (PMC2736379; doi:10.1371/journal.ppat.1000581)

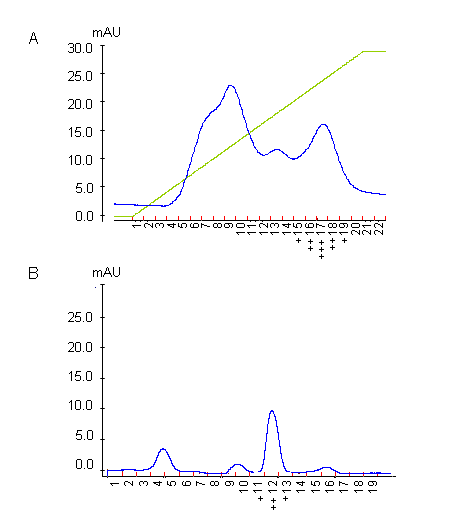

Supplement: Figure S1 — Partial purification of SnTox3 by ion exchange (A) and size exclusion (B) chromatography. PrimeView software (GE healthcare, Piscataway, NJ) was used to create chromatogram graphs with blue and green showing the instant UV absorbance, and % buffer B (300 mM NaCl), respectively. A. Ion exchange with a 0 to 300 mM NaCl gradient elution of Sn4 dialyzed culture filtrates. The Y-axis indicates the UV absorbance (blue line) and X-axis indicates the fraction number. The fractions containing SnTox3 activity are indicated by 3 plus (high activity), 2 plus (moderate activity) and 1 plus (low activity). B. Size exclusion chromatography of fraction 17 from the previous ion exchange chromatography. The Y-axis indicates the UV absorbance (blue line) and the X-axis indicates the fraction number. The fractions containing SnTox3 activity were indicated by 2 plus (moderate activity) and 1 plus (low activity). (0.02 MB TIF) [file ppat.1000581.s003.tif]

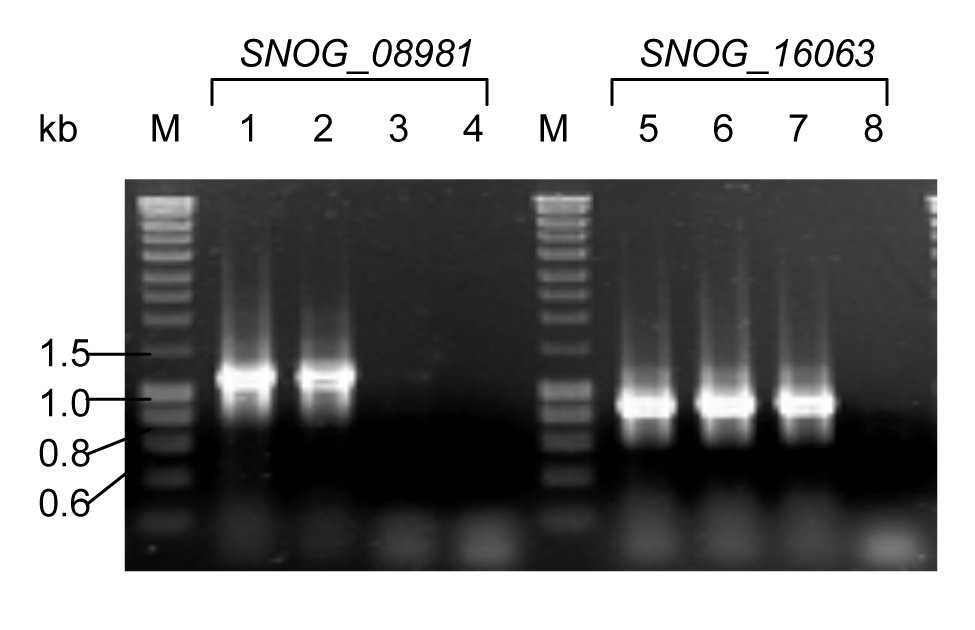

Supplement: Figure S2 — PCR testing of SNOG_08981 and SNOG_16063 identified from mass spectrometry for presence in Sn79-1087. The genomic region of two genes were amplified from Sn15 (Lane 1 and 5), Sn4 (Lane 2 and 6), Sn79-1087(Lane 3 and 7) and water controls (lane 4 and 8) using primer pairs 8981g1F and 8981g1R for SNOG_08981, 16063g1F and 16063g1R for SNOG_16063 (See Table S1 for primer sequences). Hyperladder I from Bioline was used as a size standard. The fragment size from SN15 was 1,186 bp and 824 bp for SNOG_08981 and SNOG_16063, respectively. (2.55 MB TIF) [file ppat.1000581.s004.tif]

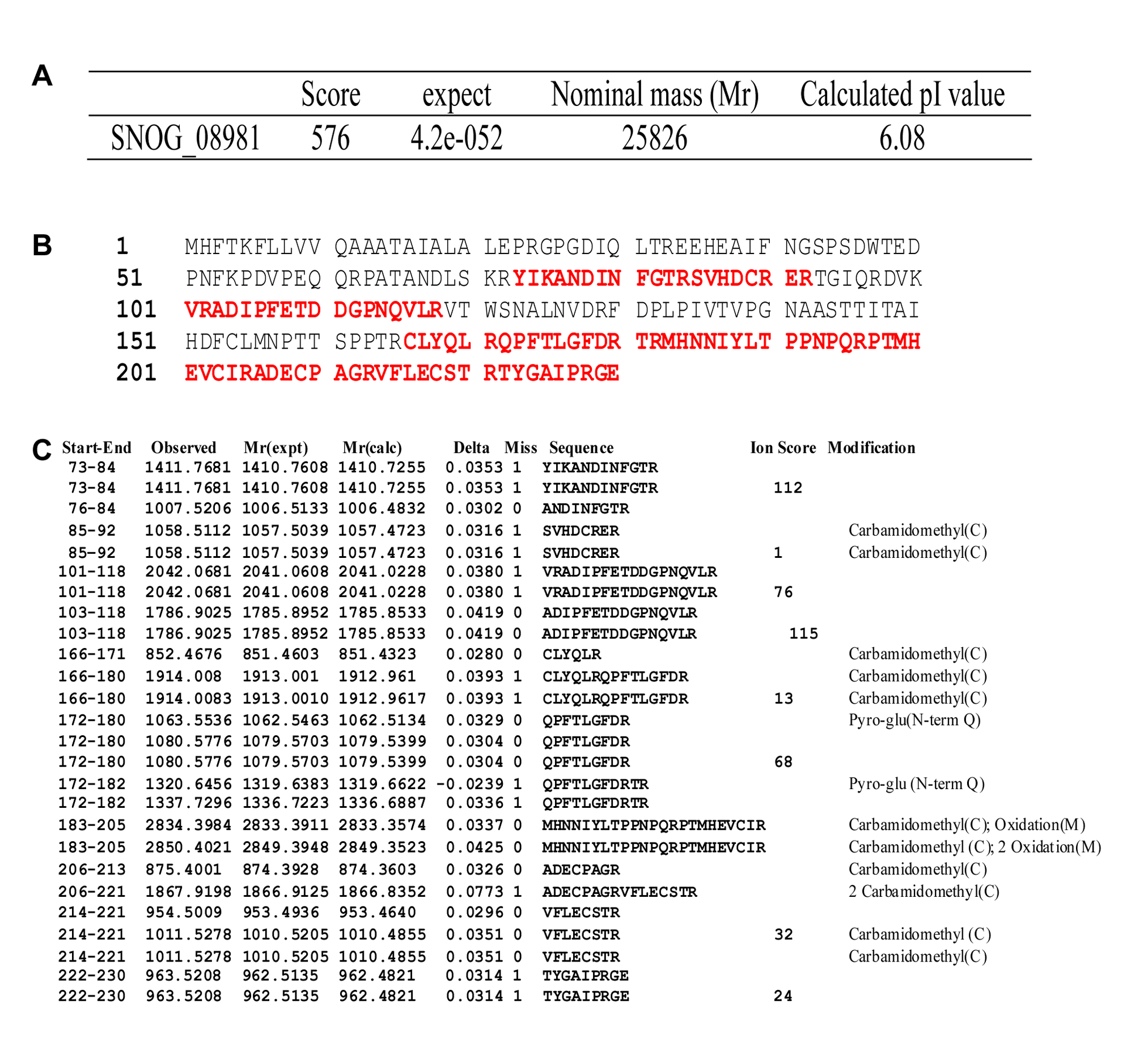

Supplement: Figure S3 — Protein view of SNOG_08981 from the mascot search report. A. Summary of SNOG_08981 in the mascot search. B. The deduced amino acid sequence of SNOG_08981. Red peptides were revealed by the mascot search. C. A list of SNOG_08981 peptide hits identified by the mascot search. (0.89 MB TIF) [file ppat.1000581.s005.tif]

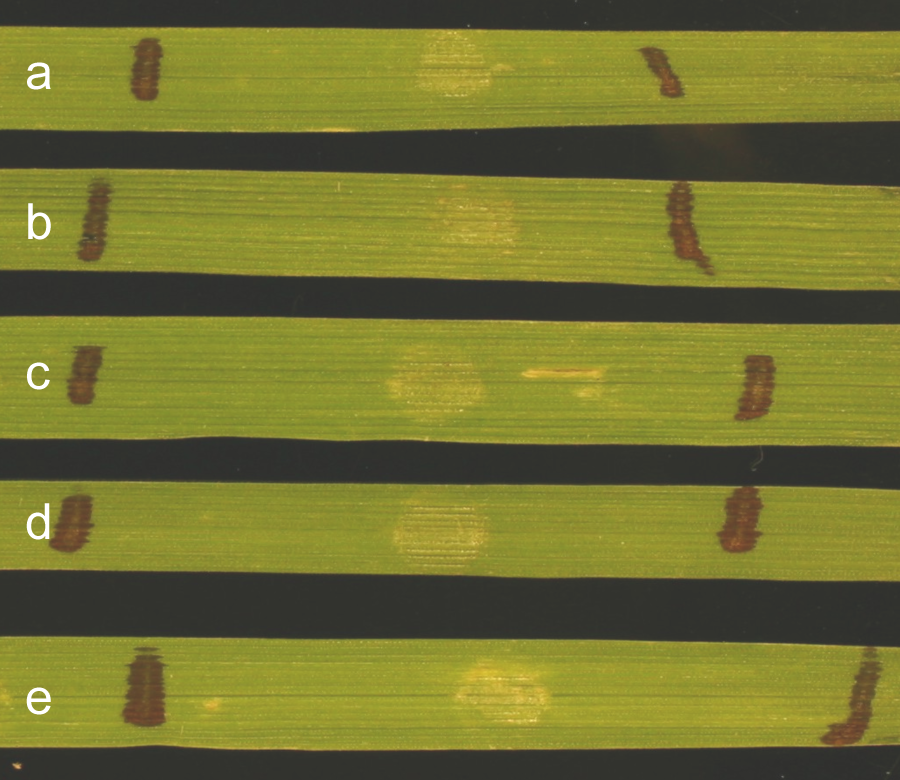

Supplement: Figure S4 — Toxin bioassay of P. pastoris transformed with an empty express vector. The leaves of BR34 (a), Grandin (b), BG261 (c), BG220 (d), and BG223 (e) were infiltrated with culture filtrates from a yeast strain transformed with an empty expression vector and photographed 3 days after infiltration. (3.82 MB TIF) [file ppat.1000581.s006.tif]

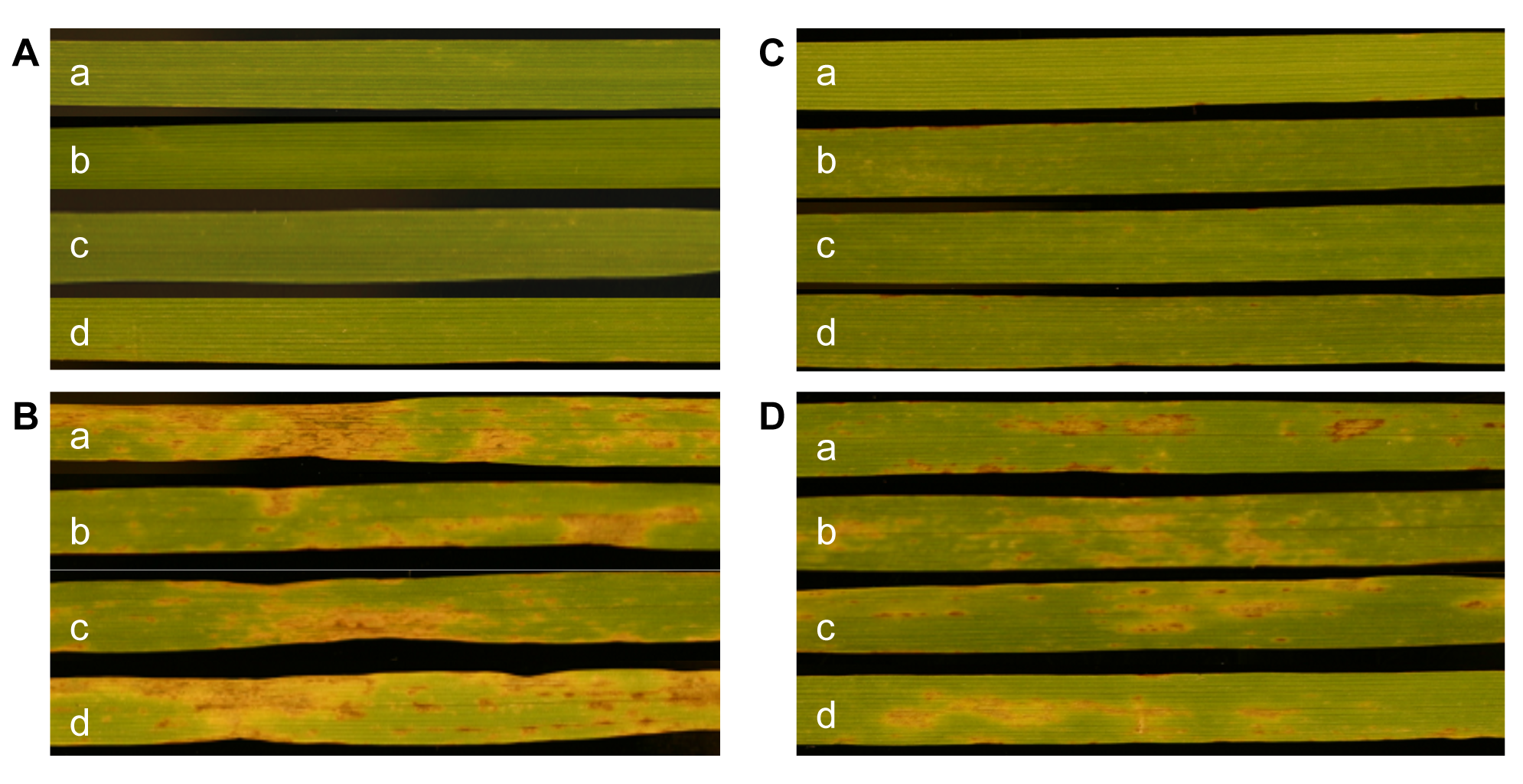

Supplement: Figure S5 — Virulence analysis of Sn1501 and its SnTox3 disrupted strains as well as an ectopic transformant on BR34, Grandin and the SnToxA and SnTox2 toxin differential lines. A. Parental line BR34 inoculated with Sn1501 wild type (a), Sn1501ΔSnTox3A (b) Sn1501ΔSnTox3B (c) and Sn1501Ect (d). All four strains show avirulence. B. Parental line Grandin inoculated with Sn1501 wild type (a), Sn1501ΔSnTox3A(b) Sn1501ΔSnTox3B(c) and Sn1501Ect(d); wild type and ectopic type strains are more virulent than the two SnTox3-disrupted strains. C. BG261 differential line (sensitive to SnToxA only) inoculated with Sn1501 wild type (a), Sn1501ΔSnTox3A(b) Sn1501ΔSnTox3B(c) and Sn1501Ect(d); all four strains are avirulent due to the absence of SnToxA in all strains. D. BG223 differential line (sensitive to SnTox2 only) inoculated with Sn1501 wild type (a), Sn1501ΔSnTox3A(b) Sn1501ΔSnTox3B(c) and Sn1501Ect(d); all four strains are equally virulent due to the presence of Snn2. (9.53 MB TIF) [file ppat.1000581.s007.tif]
